# Evaluation of the Effect of Loratadine versus Diosmin/Hesperidin Combination on Vinca Alkaloids-Induced Neuropathy: A Randomized Controlled Clinical Trial

**DOI:** 10.3390/ph17050609

**Published:** 2024-05-09

**Authors:** Noha Kamal, Mahmoud S. Abdallah, Essam Abdel Wahed, Nagwa A. Sabri, Sarah Farid Fahmy

**Affiliations:** 1 Clinical Pharmacy Department, Faculty of Pharmacy, University of Sadat City (USC), Sadat City 32897, Egypt; mahmoud.samy@fop.usc.edu.eg; 2 Department of PharmD, Faculty of Pharmacy, Jadara University, Irbid 21110, Jordan; 3 Hematology and Bone Marrow Transplantation Unit, Internal Medicine Department, Faculty of Medicine, Ain Shams University, Cairo 11591, Egypt; abdelwahed.essam@yahoo.com; 4 Clinical Pharmacy Department, Faculty of Pharmacy, Ain Shams University, African Union Organization Street, Cairo 11566, Egypt; nagwa.sabri@pharma.asu.edu.eg (N.A.S.); sarah.farid@pharma.asu.edu.eg (S.F.F.)

**Keywords:** vinca alkaloids, vincristine, neurotoxicity, neuropathy, prevention, diosmin, hesperidin, loratadine

## Abstract

Neurological injury is a crucial problem that interferes with the therapeutic use of vinca alkaloids as well as the quality of patient life. This study was conducted to assess the impact of using loratadine or diosmin/hesperidin on neuropathy induced by vinca alkaloids. Patients were randomized into one of three groups as follows: group 1 was the control group, group 2 received 450 mg diosmin and 50 mg hesperidin combination orally twice daily, and group 3 received loratadine 10 mg orally once daily. Subjective scores (numeric pain rating scale, douleur neuropathique 4, and functional assessment of cancer therapy/gynecologic oncology group–neurotoxicity (FACT/GOG-Ntx) scores), neuroinflammation biomarkers, adverse drug effects, quality of life, and response to chemotherapy were compared among the three groups. Both diosmin/hesperidin and loratadine improved the results of the neurotoxicity subscale in the FACT/GOG-Ntx score (*p* < 0.001, *p* < 0.01 respectively) and ameliorated the upsurge in neuroinflammation serum biomarkers. They also reduced the incidence and timing of paresthesia (*p* = 0.001 and *p* < 0.001, respectively) and dysuria occurrence (*p* = 0.042). Both loratadine and diosmin/hesperidin attenuated the intensity of acute neuropathy triggered by vinca alkaloids. Furthermore, they did not increase the frequency of adverse effects or interfere with the treatment response.

## 1. Introduction

Vinca alkaloids were extracted from *Catharanthus roseus* leaves of the Madagascar periwinkle plant in the late 1950s [1]. They were initially used as antidiabetic and antimalarial remedies [2]. Nowadays, vinblastine (VBL) and vincristine (VCR) are used for the management of several hematologic malignancies and solid tumors [3].

Vincristine has the greatest tendency to cause neurotoxicity among vinca alkaloids, and this subsequently results in VCR dose reduction [4]. VBL-induced neurotoxicity is less severe than neuropathy caused by VCR [5]. The incidence of peripheral neuropathy is 30–40% in patients treated with VCR [6]. Alternatively, the incidence of long-term neurological complications ranges from 50% to 97% in individuals who receive VBL [7]. Vincristine-induced peripheral neuropathy (VIPN) may potentially persist after discontinuing treatment, resulting in permanent neurological damage [8]. Early-onset VIPN could arise throughout the first chemotherapy cycle irrespective of the total VCR dose or the number of treatment cycles [9].

First-generation vinca alkaloids are accompanied by profound combined sensory/motor and autonomic neuropathy [10]. Common characteristics of sensory neuropathy include numbness, tingling, and neuropathic pain experienced bilaterally in the upper and lower extremities [11]. Common motor neuropathy signs and symptoms include footdrop and upper and lower extremity weakness [12]. Vinca alkaloids have also been linked to autonomic motor effects such as cramping in the abdomen, constipation, adynamic ileus, and urine retention [10]. While acute neuropathy may be reversible, chronic neurological damage may not be [13].

As no agent to date has proven effective in chemotherapy-induced neuropathy (CIPN) prophylaxis, the American Society for Clinical Oncology (ASCO) and the European Society for Medical Oncology (ESMO) guidelines for CIPN recommend against the use of any agent for prophylaxis against CIPN but recommend treatment utilizing duloxetine for cancer patients suffering from agonizing CIPN [14,15]. More research and development of innovative treatments is crucial because vinca alkaloids-induced neuropathy can have substantial repercussions on the quality of patient life and clinical outcomes [16].

In a double-blinded clinical trial, diosmin/hesperidin ameliorated oxidative stress in patients with type 1 diabetes resulting in improved diabetic neuropathy symptoms [17]. There is compelling evidence that diosmin/hesperidin protects against neuropathy and reduces neuropathic pain in animal models [18,19,20,21]. Diosmetin, the main bioactive metabolite of diosmin, reduced calcium influx through transient receptor potential vanilloid 1 (TRPV1) and produced antinociceptive and anti-inflammatory effects in male mice [19]. Diosmin also decreased neuronal injury and improved perception in a mouse model [20]. Hesperidin stimulated peroxisome proliferator-activated receptor gamma (PPAR-γ), which inhibited the nuclear factor kappa B (NF-κB) and mitogen-activated protein kinase (MAPK) activation pathways [21]. Loratadine also showed promising results in the mitigation of neuropathic pain and inflammation [22]. Loratadine substantially decreased the production of genes associated with inflammation, inhibited NF-κB, and blocked activating protein-1 (AP-1) stimulation via MAPK [23]. Loratadine binds to H1-receptors in different cells and decreases the activation of peripheral nociceptive receptors [24]. Using H1 receptor antagonists causes mild analgesic activity [25]. Loratadine significantly reduced neuropathic pain induced by peripheral axotomy in rats [26]. A study that investigated a non-selective histamine blocker in a rat model recommended using a selective H1 blocker for managing VIPN [27]. Loratadine also reduced myalgia induced by paclitaxel and bone pain caused by pegfilgrastim in patients with cancer [28,29]. Additionally, both loratadine and diosmin/hesperidin ameliorated neuropathic pain by reducing inflammation [21,22,27].

Subjective assessment scores have been employed in earlier research to evaluate neurological function in pediatric patients with cancer suffering from VIPN [11]. Numeric pain rating scale (NS) scores were reduced in patients responding to different treatments of CIPN [30]. Also, douleur neuropathique 4 (DN4) scores were increased in patients experiencing neuropathic pain because of oxaliplatin [31]. Similarly, the functional assessment of cancer therapy/gynecologic oncology group–neurotoxicity (FACT/GOG-Ntx) scores were used to assess chemotherapy-induced neuropathy and patient quality of life for women with ovarian cancer, and it showed sensitivity to the development and progression of neurotoxicity [32]. Furthermore, a previous study demonstrated that paclitaxel and docetaxel were determined to have a lower risk for CIPN than nab-paclitaxel by comparing FACT/GOG-Ntx scores [33].

The severity of neurotoxicity has been evaluated in several studies by measuring serum biomarkers (interleukin 1-beta (IL1-β), tumor necrosis factor-alpha (TNF-α), neurofilament protein light polypeptide (NFL), and nerve growth factor (NGF)) at baseline and after three cycles of vinca alkaloids therapy [34,35,36,37]. The increase in IL1-β and TNF-α levels has been used for the detection of the neurotoxicity of VCR and paclitaxel [34,38]. The overexpression of both markers is associated with inflammation and a higher response to nociceptive stimuli [35]. Serum levels of NFL increased with higher levels of paclitaxel-induced neuropathy severity [39]. The sustained administration of oxaliplatin contributed to a gradual rise in NFL levels from baseline, as well as after 3 and 6 months [36]. Additionally, the level of NGF was used for the detection of nerve damage in several studies [37,40,41].

No clinical trial to date has been conducted to assess the effect of either diosmin/hesperidin or loratadine on the prevention of neuropathy triggered by vinca alkaloids despite their promising outcomes. Therefore, this study investigated the potential impact of loratadine and diosmin/hesperidin as prophylactic agents against neurotoxicity associated with vinca alkaloids therapy.

## 2. Results

### 2.1. Patient Demographics and Clinical Characteristics

**Figure 1:**
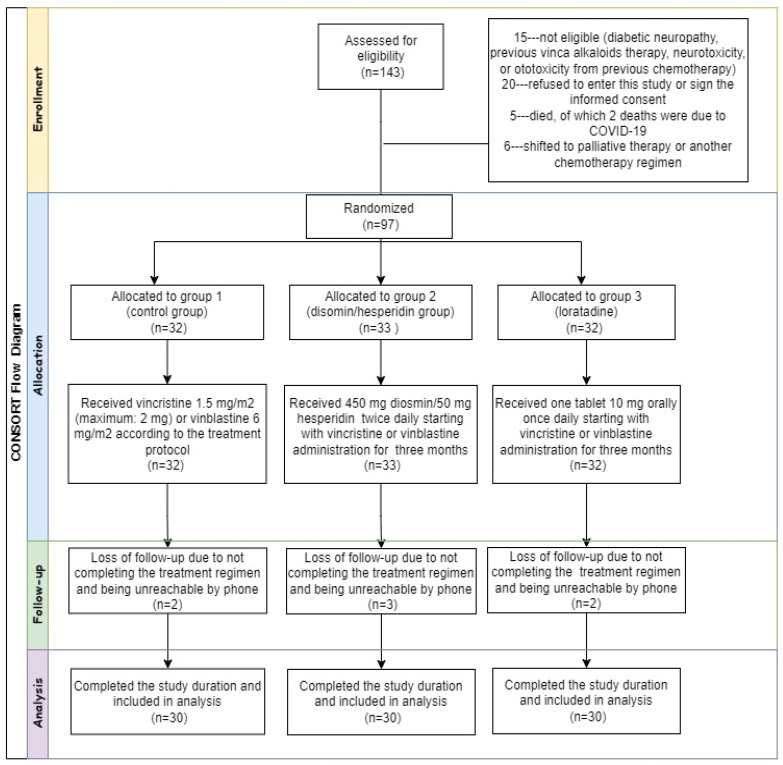
Consolidated standards of reporting trials (CONSORT) flow diagram.

Patient recruitment was carried out following the inclusion and exclusion criteria established in the study design prior to the start of the investigation. The allocation of patients to the three groups is illustrated in the consolidated standards of reporting trials (CONSORT) flow diagram (Figure 1). Table 1 shows the demographics and clinical features of the three groups of patients.

The three groups had no statistically significant difference in baseline characteristics and clinical features except for weight and body surface area (BSA), as both showed significant increases in group 2. However, this difference did not affect the vinca alkaloids dose as VCR and VBL had a maximum of 2 mg per dose and 10 mg per dose, respectively. After performing both multivariate and univariate regression analyses for every variable, it was found that weight and BSA did not modify any of the primary or secondary outcome variables. All patients included in this study suffered from hematological cancers and received vinca alkaloids therapy, as represented in Table 2. 

### 2.2. Biochemical Tests for the Three Groups at Baseline and after the End of Every Cycle of Vinca Alkaloid Chemotherapy

In terms of safety, diosmin/hesperidin and loratadine had no negative impact on kidney or liver functions or blood cell counts compared to the control group.

Kidney function tests showed no statistically significant difference among the three groups, as presented in Supplementary Table S1. Blood urea nitrogen (BUN) levels increased in group 1. On the other hand, group 3 demonstrated an increase in BUN levels and estimated glomerular filtration rate (eGFR) values but a decline in serum creatinine levels compared with the baseline. 

The liver function tests revealed non-statistically significant differences among groups, as shown in Supplementary Table S2. In group 2, total bilirubin decreased compared with baseline levels and showed the lowest levels of aspartate aminotransferase (AST) and alanine aminotransferase (ALT) levels after the end of the third cycle. In group 3, direct bilirubin levels were lower than group 1 levels after the end of the first cycle. Blood cell counts improved in the three groups with treatment, as presented in Supplementary Table S3. 

### 2.3. Assessment of Vinca Alkaloid-Induced Neuropathy Results

#### 2.3.1. Subjective Assessment Scores at Baseline and after Treatment

For NS assessment, the patients were asked to rate the level of their pain across the vinca alkaloids cycle from “0” (no pain) to “10” (worst pain) with every neuropathy-related adverse effect. DN4, a French questionnaire with the English translation “neuropathic pain in four questions, comprises four questions divided into ten items based on sensory descriptors and sensory examination signals. The investigator assigned one point to each positive response. The overall score was computed using the sum of the ten components.

Non-significant differences were found among the three groups in NS and DN4 scores in the three cycles. However, the scores were lower in both groups 2 and 3 than in group 1, as represented in Table 3. In the third cycle, DN4 scores decreased (adjusted *p* = 0.029) in group 2 compared with group 1. Pairwise comparisons showed that NS scores increased from baseline to cycles 1 and 2 in the three groups, as shown in Supplementary Figure S1.

The neurotoxicity subscale of FACT/GOG-Ntx scores assesses peripheral neuropathy symptoms such as sensory, motor, and auditory difficulties and cold sensitivity. To evaluate the secondary endpoint of the safety of the use of interventions, other subscales of FACT/GOG-Ntx scores including physical, social, emotional, or functional well-being were evaluated. There was no difference in the secondary endpoint of safety in terms of the FACT/GOG-Ntx subscale scores including physical, social, emotional, functional well-being, and total scores, as presented in Supplementary Table S4. However, all subscale scores decreased in the three groups compared with the baseline and across the cycles of chemotherapy, as illustrated in Supplementary Figures S2–S7. 

The neurotoxicity subscale scores as a primary endpoint of the severity of neuropathy decreased in the three groups, but the greatest decline was in group 1, as shown in Table 3. The decrease in neurotoxicity subscale scores in group 1 was due to patients reporting worse outcomes in the questions pertinent to motor neuropathy. These questions are as follows: “I feel weak all over”, “I have trouble walking”, “I feel discomfort in my feet”, and “I feel discomfort in my hands.” Neurotoxicity subscale scores did not differ between group 2 and group 3. 

#### 2.3.2. Serum Biomarker Levels at Baseline and after Treatment

**Figure 2:**
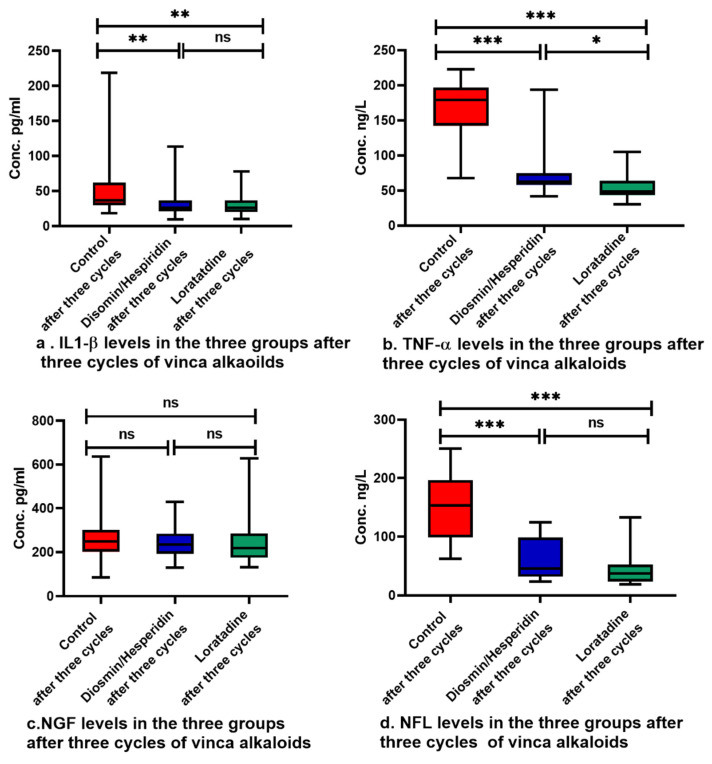
Serum biomarker levels in the three groups after treatment. Pairwise comparisons were performed utilizing Dunn’s correction. Data are expressed as median with range, ns: not statistically significant, *: *p* < 0.05, **: *p* < 0.01, ***: *p* < 0.001.

The three groups showed no statistically significant difference in the baseline levels of IL1-β, TNF-α, NGF, and NFL, as shown in Table 4. There was a significant increase in IL1-β, TNF-α, and NFL levels in the three groups, but loratadine and diosmin/hesperidin-treated patients showed lower levels of these biomarkers compared with the control group, as shown in Figure 2a,b,d. On the other hand, NGF did not change significantly from baseline, as shown in Table 4, and showed comparable levels in the three groups after treatment, as demonstrated in Figure 2c. Within the three groups, the levels of neurotoxicity biomarkers increased in the three groups, although this rise was smaller in both groups 2 and 3 compared with group 1. 

#### 2.3.3. Treatment-Induced Adverse Drug Effects 

Neuropathy-related adverse effects are shown in Table 5. The other adverse drug effects encountered by patients in this study are shown in Supplementary Table S5.

**Figure 3:**
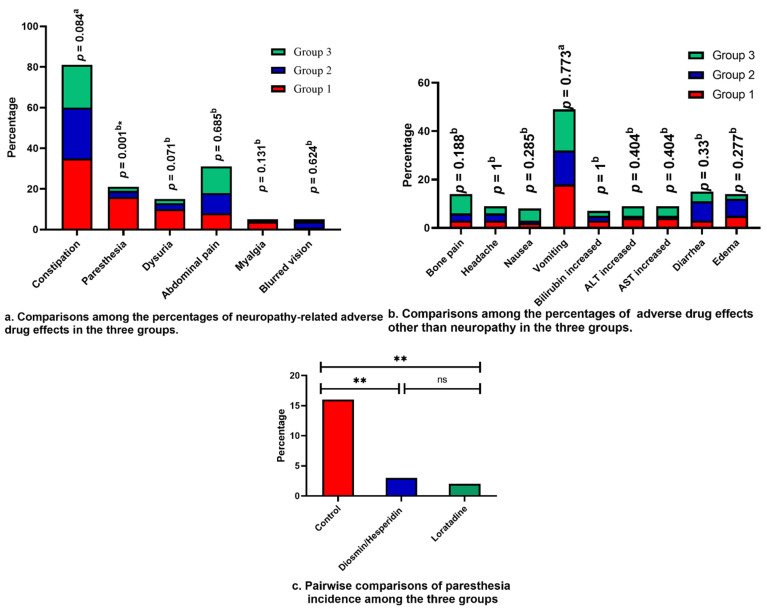
Comparisons among the percentages of adverse drug effects in the three groups. Comparisons were performed utilizing the ^a^: Chi-Square test or ^b^: Fischer exact test, ns: not statistically significant, *: *p <* 0.05, **: p < 0.01, and the data are expressed as percentages.

No significant differences were observed among the three groups in terms of the severity of adverse effects, as described by CTCAE. Grade 3 was the most severe adverse effect encountered in this study. The frequency of adverse effects did not differ significantly among the three groups except for paresthesia. The percentage of patients suffering from paresthesia was lower in both groups 2 and 3 compared with group 1. Constipation was the most frequent neuropathy-related adverse effect, while vomiting was the most common one among the other adverse effects, as illustrated in Figure 3a,b. Utilizing Fischer’s exact test, pairwise comparisons for paresthesia were performed, as shown in Figure 3c.

#### 2.3.4. Neuropathy-Related Adverse Drug Effects Timing

Neuropathy-related adverse drug effects were monitored as a primary outcome and their timing as a secondary outcome starting from the first day of vinca alkaloids administration and continued for three cycles. Patients were asked three times weekly about any adverse effects encountered during the treatment. The definitions of adverse effects that happened in this study are illustrated in Supplementary Table S6. Neuropathy-related adverse effect free time for constipation was higher in groups 2 and 3 compared with group 1, though this was not statistically significant. There was also a non-significant decrease in abdominal pain-free time in group 3 compared with the other groups and blurred vision in group 2 compared with the other groups. For paresthesia and dysuria, there was a significant increase in adverse effect free time in group 2 and group 3 compared with group 1. Cumulative survival, pairwise comparisons, hazard ratios, and adverse effect free time are presented in Supplementary Table S7, Figures S8 and S9.

### 2.4. Response to Vinca Alkaloids after Treatment

MRD was measured at baseline and after the end of the third cycle of vinca alkaloids treatment. MRD was measured by flow cytometry, where a negative MRD response was defined as less than 0.01% MRD cells (10^−4^) [42]. At baseline, all patients had positive MRD except for one patient suffering from B-cell lymphoma in group 3. After the end of the third cycle, there was no difference (*p* = 0.861) in the percentage of MRD-negative patients in groups 2 (70%) and 3 (66.7%) compared to group 1 (63.3%).

### 2.5. Encountered Drug–Drug Interactions during Therapy

Drug-drug interactions were checked through the Lexicomp^®^ interaction checker [43]. Noteworthy drug–drug interactions encountered during the study period were documented and reported (Supplementary Table S8).

The reported drug–drug interactions included voriconazole interaction with vinca alkaloids, which makes it an unfavorable antifungal choice for patients receiving vinca alkaloids [44,45]. In addition, fluconazole requires monitoring for increased vinca alkaloids toxicities [46]. Both drugs increase the serum levels of vinca alkaloids [43,46]. It is preferred to discontinue azole antifungals for 24 hours preceding the infusion of vinca alkaloids and resume them 24 hours afterward [45]. If antifungal therapy is necessary, an echinocandin (for example, micafungin, caspofungin, or anidulafungin) could substitute for the azoles [45]. 

### 2.6. Correlation Analysis

To determine if serum neuropathy scores were correlated with the serum levels of biomarkers and other neuropathy scores after treatment, linear regression studies with Spearman’s correlation were performed (Supplementary Figure S10). Both NS and DN4 scores showed considerable correlation with TNF-α levels. Patients with higher NS scores showed inferior performance, as indicated by lower NTX subscale in FACT/GOG-Ntx scores. IL-β increased in correlation with TNF-α and NFL levels. Higher NGF at baseline resulted in higher levels after treatment. However, patients with lower NGF levels had higher levels of TNF-α.

## 3. Discussion

The development of neuropathy poses a challenge to the therapeutic use of vinca alkaloids [47]. Therefore, the development of preventive strategies is essential. Despite the encouraging findings of loratadine, diosmin, and hesperidin in prior studies, no clinical study has been completed so far to evaluate their effect on vinca alkaloids neuropathy.

Diosmin/hesperidin alleviated neuropathy symptoms. It resulted in lower NS and DN4 scores compared with the control group, but the difference was not statistically significant. Comparable results were found in rats as diosmin/hesperidin reduced neuropathic pain, hyperalgesia, and inflammation and protected against diabetic neuropathy [18]. 

In the current study, the neurotoxicity subscale in FACT/GOG-Ntx scores revealed milder motor neuropathy with diosmin/hesperidin or loratadine compared with the control group. In a similar manner, diosmin protected against neuropathy induced by scopolamine in a rat model [48]. Diosmin inhibited arsenic-induced neurotoxicity in rats as well [49]. Likewise, hesperidin administration for 90 days improved cognitive problems in mice [50]. Furthermore, hesperidin protected *Drosophila melanogaster* male adult flies against iron-induced neurotoxicity [51], and it demonstrated neuroprotection in animal studies of neurotoxicity and chronic neurodegenerative disorders, presenting it as an intriguing yet harmless dietary compound to be explored further as a supplemental remedy [52]. In addition, the use of desloratadine, an active metabolite of loratadine, improved the viability of mouse embryonal carcinoma P19 cells (P19 neurons), which makes the utilization of antihistaminic medications for neuronal injury an interesting field of research [53].

Diosmin/hesperidin and loratadine groups showed reduced inflammatory markers including IL-β, TNF-α, and NFL levels. The higher inflammation suggests a higher severity of neuropathy in the control group. Similar to our findings, diosmin reduced TNF-α, which was elevated as a result of nerve damage [48]. Hesperidin also alleviated neuroinflammation through the reduction of IL1-β and TNF-α in patients with type 2 diabetes [54]. Loratadine also diminished inflammatory mediators such as IL1-β and TNF-α through the inhibition of NF-κB in a mouse model [23]. Additionally, loratadine, when combined with montelukast, resulted in a reduction in IL-4 and TNF-α without increasing adverse effects in children with asthma [55]. In a comparable manner, thalidomide and minocycline decreased paclitaxel-induced neuropathic pain in rats through TNF-α and IL1-β suppression [56]. Moreover, hesperidin reduced the level of serum neurofilament in induced spinal cord injury in a rat model [57]. The level of NGF was consumed because of nerve injury in many studies [37,40,58]. However, other studies revealed a rise in NGF levels because of neuronal damage or inflammation [41,59,60]. NGF levels did not differ from baseline and were comparable among the three groups in the present research. However, the control group showed slightly lower levels than the intervention groups. This may be due to endogenous neurotrophin elevation during nerve regeneration in the axotomized neurons [61]. NGF was also increased during the recovery of nerve injury in a rat model [62]. 

In terms of the secondary endpoint of safety, the FACT/GOG-Ntx score subscales including physical, social, emotional, or functional well-being were not altered by either diosmin/hesperidin or loratadine. This implies that they did not adversely affect the QOL of patients. Similar to this, FACT/GOG-Ntx scores were utilized to assess the safety of Yoga in survivors of cancer suffering from CIPN [63]. 

In terms of neuropathy symptoms, diosmin/hesperidin and loratadine reduced the incidence of paresthesia and the timing of both paresthesia and dysuria. Both medications were safe as they did not increase the incidence of non-neuropathy-related adverse effects. The number of patients experiencing constipation was not significantly lower than the control group for patients receiving either diosmin/hesperidin or loratadine. In contrast with these findings, a single administration of either hesperidin or diosmin decreased hyperalgesia in rat models [18,64]. This effect was enhanced by using a combination of both agents [65]. Hesperidin increased both motor and sensory nerve conduction velocity, and it also improved behavioral and cognitive outcomes in mice [50,66]. A comparable set of outcomes was observed with loratadine as it reduced the rate of symptoms of myalgias and arthralgias from 70% to 34% in patients receiving paclitaxel [67]. Several studies demonstrated that loratadine prophylaxis was a safe choice for mitigation of the bone pain induced by pegfilgrastim [22,28,29].

Concerning the safety of interventions, they did not modify MRD values that were utilized as a measure of the patient response to chemotherapy in the current investigation. This is inconsistent with previous study results indicating that skin cancer cell growth in rats was inhibited by diosmin [68]. Human glioblastoma cell death was also enhanced by diosmin [69]. Loratadine also inhibits transforming growth factor-β-activated kinase 1 (TAK1), which plays a substantial role in apoptosis, making it an appropriate target for the treatment of a variety of conditions including cancer [23]. This discrepancy could be attributed to the assessment of the effect of these medications on different types of cancer including solid tumors in the skin, brain, ovary, and cervix. However, all the patients in the current study suffered from cancers of hematological origin.

A further crucial aspect of safety is the effect of interventions on kidney function. Surprisingly, loratadine administration resulted in an improvement in eGFR in the current study. This improvement could indicate that loratadine protected the kidney against malignancy or chemotherapy-induced nephrotoxicity. Similar to this outcome, a retrospective cohort of 1467 patients with cancer revealed that patients who received diphenhydramine, an H1 antagonist, prior to cisplatin administration had a lower incidence of acute kidney injury (6.1%) compared with patients who did not receive it (22.4%) [70]. In this research, the diosmin/hesperidin group also exerted beneficial effects on the kidneys, as it exhibited stable BUN levels in contrast to slowly rising values in the other groups. In a rat model, diosmin protected against doxorubicin-induced nephrotoxicity by reducing inflammatory markers such as IL1-β and TNF-α and increasing glutathione levels [71].

Regarding liver function tests, patients receiving diosmin/hesperidin demonstrated lower levels of bilirubin, AST, and ALT compared with the other groups in this trial. Similarly, diosmin protected from cholestasis and liver damage in rats by downregulating NF-κB, MAPK, Kelch-like ECH-associating protein 1 (Keap-1), and inducible nitric oxide synthase (iNOS) [72]. Hesperidin also ameliorated hepatic cell injury resulting from nano zinc oxide particles and reduced AST, ALT, and oxidative stress in a rat model [73].

A thorough review would reveal that there is compelling proof indicating that both diosmin/hesperidin and loratadine have promising outcomes. Studies on hesperidin and diosmin have revealed a range of protective potentials, including neuroprotection, hepatoprotection, suppressing inflammatory processes, and diminishing oxidative stress, with no symptoms of toxicity [74]. Loratadine is also an extensively prescribed anti-histaminic medication whose effectiveness and safety have been successfully authorized by the Food and Drug Administration (FDA) [23].

The main study limitation of this study is the open-label design, although laboratory investigators were uninformed of the assignment of patients to study groups during biochemical analysis. It is preferable to utilize a blinded placebo-controlled approach to improve the validity of the results. In addition, the follow-up period of a three-month duration is relatively short. Future studies should adopt a double-blinded design and longer follow-up periods to assess the effectiveness and safety of intervention medications. 

## 4. Materials and Methods 

### 4.1. Study Design and Setting

This study was a prospective, controlled, randomized, and open-label clinical trial. This research was conducted from February 2022 to May 2023. The last patient was enrolled in February 2023 and completed follow-up through May 2023. It was conducted in the Hematology Department at Ain Shams University Hospitals.

### 4.2. Ethical Consideration

This study was conducted in compliance with the Declaration of Helsinki guidelines. It was approved by the ethical committee of the Faculty of Pharmacy, Ain Shams University (approval number: ACUC-FP-ASU RHDIRB2020110301 REC#71), and the research ethics committee of the Faculty of Medicine, Ain Shams University (approval number: FMASU UNIV 2/2022). All the patients signed an informed consent form without any obligation, and they were free to withdraw at any time during treatment. The study protocol was registered on clinicaltrials.gov (NCT05243706).

### 4.3. Patients

All patients prescribed vinca alkaloids for the management of hematological malignancies were assessed for eligibility. Patients were included in this study if they were prescribed VCR or VBL according to standard protocols, were 18 to 70 years old with an eastern cooperative oncology group (ECOG) performance status grade 0–2, were willing to participate in this study, and signed the informed consent.

Patients were excluded from this research if they had any of the subsequent criteria: received previous vinca alkaloids therapy, hypersensitivity or contraindication to loratadine, hesperidin/diosmin combination or any component of the formulation, pre-existing or history of peripheral neuropathy, receiving any medication known to cause neuropathy, receiving medications with drug interaction grade X with loratadine as thalidomide, tiotropium, or orphenadrine, severe hepatic impairment, or inability to understand patient information and the informed consent form.

Patients who met the eligibility criteria were randomly assigned to one of three groups. The randomization list was generated utilizing the website http://www.jerrydallal.com/random/random_block_size_r.htm accessed on 15 October 2021. Group 1 (control group) received VCR 1.5 mg/m^2^ (maximum: 2 mg) or VBL 6 mg/m^2^ (maximum 10 mg) according to the treatment protocol. Group 2 (diosmin/hesperidin group) received one film-coated tablet of 450 diosmin and 50 mg hesperidin micronized purified flavonoid fraction (MPFF) combination orally twice daily starting with VCR or VBL administration and continued for a three-month duration [75]. Group 3 (loratadine group) received one tablet of 10 mg loratadine orally once daily starting with VCR or VBL administration and continued for a three-month duration [28].

### 4.4. Methods

The primary outcomes of this study were the development and the severity of vinca alkaloids neuropathy in the three groups. The secondary outcomes included the timing of neuropathy caused by vinca alkaloids as well as the safety of loratadine and diosmin/hesperidin in terms of undesirable effects. Kidney function tests, liver function tests, and blood cell counts were all monitored at baseline and at the end of every cycle of vinca alkaloids for three cycles to ensure safety. Moreover, adverse drug effects, quality of life (QOL), and response to vinca alkaloids were evaluated among the three groups.

All patients’ full histories including age, sex, medical history, concurrent diseases, and concurrent medications were gathered and documented in a pre-prepared follow-up sheet. For the assessment of primary outcomes, the severity was evaluated clinically using the Arabic-validated versions of the NS, DN4, and FACT/GOG-Ntx scores. For three cycles of vinca alkaloids therapy, the questionnaires were deployed at baseline and at the end of every cycle.

Adverse drug effects were described utilizing the National Cancer Institute (NCI) common terminology criteria for adverse events (CTCAE) version 5.0 published on 27 November 2017 [76]. 

The severity of neurotoxicity as a primary outcome was also evaluated biochemically by measuring serum biomarkers (IL1-β, TNF-α, NFL, and NGF) at baseline and after three cycles of vinca alkaloids therapy.

For further monitoring of intervention safety, patients were monitored for the response to vinca alkaloids treatment by following measurable residual disease (MRD; previously termed minimal residual disease) [42]. 

### 4.5. Sample Size Calculation

The sample size was calculated assuming a relatively large effect size as proven by previous studies on animals [65,77]. In a one-way ANOVA study, sample sizes of 25 patients per group in the three groups—totaling 75 patients—were required for comparing means. This achieved 80% power using an F test with a 0.05 significance level. The size of the variation in the means was represented by the effect size f = σm/σ, which is 0.40. An extra 25% was added to the calculated sample size to boost the power and precision of the results, so ninety patients were enrolled. 

### 4.6. Statistical Analysis

Analyses and graphics were performed utilizing the International Business Machines Corporation statistical package for social sciences (IBM SPSS version 22, New York, NY, USA), GraphPad Prism 8.0.2.236 version (GraphPad Software, San Diego, CA, USA), and Microsoft Excel 2010. The expressions of data were provided as mean ± SD for continuous normally distributed data, median (interquartile range (IQR) for not normally distributed, and frequency in tables for categorical data. Kolmogorov–Smirnov and Shapiro–Wilk tests were performed to check normality before performing any analysis. Comparisons among the three groups for quantitative parametric values were performed using ANOVA tests, while non-parametric values were tested utilizing the Kruskal–Wallis test. Post hoc analysis was corrected using the Bonferroni correction for ANOVA tests and using Dunn’s test for Kruskal–Wallis tests. Categorical variables and proportions were compared using the Chi-square test. Fisher’s exact test was used if any of the cells had expected counts less than 5. The timing of the development of adverse drug effects was compared using Kaplan–Meier analysis, while hazard ratios were calculated utilizing the Cox regression model. Correlations were estimated by performing Spearman rank correlation analyses. Adjusted two-tail *p*-value < 0.05 was considered significant.

## 5. Conclusions

The outcomes of this study suggest that both loratadine and diosmin/hesperidin attenuated the severity of acute neuropathy associated with vinca alkaloids. This was manifested clinically by higher neurotoxicity subscale scores in FACT/GOG-Ntx scores and biochemically by a lower rise in TNF-α, IL-β, and NFL serum levels. The intervention drugs had the greatest influence on sensory and motor neuropathy resulting from vinca alkaloids. They exhibited no harmful influence on the side effects or response to therapy. This renders both loratadine and diosmin/hesperidin safe and tolerable choices for further investigation of prophylaxis against vinca alkaloids-associated toxicities.

## Figures and Tables

**Table 1:** Patients’ demographics and clinical characteristics of the three groups at baseline.

Demographics	Control (Group 1) *n* = 30	Diosmin/Hesperidin (Group 2) *n* = 30	Loratadine (Group 3) *n* = 30	*p*-Value
Age (mean ± SD), years	35.33 ± 13.85	40.5 ± 15.42	41.93 ± 17.67	0.237 ^a^
Gender				
Male [*n*, (%)]	17 (56.7%)	20 (66.7%)	20 (66.7%)	0.760 ^b^
Female [*n*, (%)]	13 (43.3%)	10 (33.3%)	10 (33.3%)	
Diabetes [*n*, (%)]	2 (6.7%)	3 (10.0%)	2 (6.7%)	0.999 ^c^
Hypertension [*n*, (%)]	0 (0%)	4 (13.3%)	3 (10.0%)	0.154 ^c^
Smoking [*n*, (%)]				
Ex-smoker	1 (3.3%)	1 (3.3%)	1 (3.3%)	
Non-smoker	23 (76.7%)	19 (63.3%)	23 (76.7%)	0.634 ^c^
Smoker	6 (20.0%)	10 (33.3%)	6 (20.0%)	
Weight (mean ± SD), Kg	68.67 ± 12.52	80.83 ± 19.09	72.1 ± 14.48	0.010 ^a^*
Height, (mean ± SD), cm	168.37 ± 8.38	169.83 ± 9.51	168.5 ± 9.3	0.787 ^a^
BSA (mean ± SD), cm^2^	1.78 ± 0.17	1.94 ± 0.26	1.83 ± 0.21	0.017 ^a^*
ECOG performance status [median, (IQR)]	0 (0–1)	0 (0–1)	1 (0–1)	0.5229 ^d^

^a^: One-way ANOVA used as a statistical test, ^b^: Chi-square test, ^c^: Fisher’s exact test, ^d^: Kruskal–Wallis test, *: a statistically significant difference. Normally distributed data values are expressed as mean ± standard deviation; for all statistical tests used, a *p*-value < 0.05 is considered statistically significant. BSA: body surface area, ECOG: Eastern Cooperative Oncology Group, IQR: interquartile range.

**Table 2:** Types of cancer, chemotherapy protocols, and vinca alkaloids dose in the three groups.

Parameters	Control (Group 1) *n* = 30	Diosmin/Hesperidin (Group 2) *n* = 30	Loratadine (Group 3) *n* = 30	*p*-Value
Types of cancer: [*n*, (%)]				
B-Cell lymphoma	0 (0.0%)	0 (0.0%)	1 (3.3%)	
B-ALL	11 (36.7%)	10 (33.3%)	12 (40.0%)	
Burkitt lymphoma	1 (3.3%)	0 (0.0%)	2 (6.7%)	
CLL	3 (10.0%)	2 (6.7%)	4 (13.4%)	
HLH	0 (0.0%)	0 (0.0%)	1 (3.3%)	0.480 ^a^
Hodgkin lymphoma	3 (10.0%)	2 (6.7%)	1 (3.3%)	
Marginal cell lymphoma	1 (3.3%)	0 (0.0%)	0 (0.0%)	
Multiple myeloma	1 (3.3%)	4 (13.3%)	0 (0.0%)	
NHL	1 (3.3%)	6 (20.0%)	4 (13.3%)	
Plasma cell myeloma	1 (3.3%)	0 (0.0%)	0 (0.0%)	
T-Cell lymphoma	1 (3.3%)	1 (3.3%)	0 (0.0%)	
T-ALL	7 (23.3%)	5 (16.7%)	5 (16.7%)	
Chemotherapy Protocols: [*n*, (%)]				
ABVD	3 (10.0%)	2 (6.7%)	1 (3.3%)	
C-VAMP	2 (6.7%)	4 (13.3%)	0 (0.0%)	
CALGB	2 (6.7%)	2 (6.7%)	4 (13.3%)	
CHEOP	0 (0.0%)	0 (0.0%)	1 (3.3%)	
CHOP	1 (3.3%)	2 (6.7%)	0 (0.0%)	0.469 ^a^
COP	3 (10.0%)	4 (13.3%)	6 (20.0%)	
HCVAD-A	18 (60.0%)	13 (43.3%)	13 (43.3%)	
R-CHOP	1 (3.3%)	3 (10.0%)	4 (13.3%)	
VAD	0 (0.0%)	0 (0.0%)	1 (3.3%)	
Vinca alkaloids dose:				
Vincristine [median, (IQR)]	4 (2–8)	4 (2–8)	4 (2–8)	0.5234 ^b^
Vinblastine	20 (18–20)	19.5 (19–20)	20	

^a^: Fisher’s exact test. ^b^: Kruskal–Wallis test. For all statistical tests used, *p*-value < 0.05 is considered statistically significant. ABVD: doxorubicin, bleomycin, vinblastine, and dacarbazine. B-ALL: acute B lymphoblastic leukemia. C-VAMP: cyclophosphamide, vincristine, doxorubicin, and methylprednisolone. CALGB: daunorubicin, vincristine, prednisone, asparaginase, and cyclophosphamide. CHEOP: cyclophosphamide, doxorubicin, vincristine, etoposide, and prednisolone. CHOP: cyclophosphamide, doxorubicin, vincristine, and prednisolone. CLL: chronic lymphocytic leukemia. COP: cyclophosphamide, vincristine, and prednisone. HCVAD-A: modified hyper-fractionated cyclophosphamide, vincristine, doxorubicin, and dexamethasone. HLH: hemophagocytic lympho-histiocytosis. IQR: interquartile range. NHL: non-Hodgkin lymphoma. R-CHOP: rituximab, cyclophosphamide, doxorubicin, vincristine, and prednisolone. T-ALL: acute T lymphoblastic leukemia. VAD: vincristine, doxorubicin, and dexamethasone.

**Table 3:** Subjective assessment scores at baseline and the average across the three cycles of vinca alkaloids in the three groups.

Score	Assessment Time	Control (Group 1) *n* = 30	Diosmin/Hesperidin (Group 2) *n* = 30	Loratadine (Group 3) *n* = 30	*p*-Value
NS	At baseline	0	0	0	1 ^a^
	Average of first cycle	2 (0–2)	2 (0–2)	0 (0–2)	0.670 ^a^
	Average of second cycle	1 (0–3)	0 (0–2)	0 (0–2)	0.141 ^a^
	Average of third cycle	0.5 (0–2)	0 (0–1)	0 (0–2)	0.058 ^a^
	*p*-value	<0.001 ^b^*	<0.0001 ^b^*	0.0003 ^b^*	
DN4	At baseline	0	0	0	1 ^a^
	Average of first cycle	0 (0–1)	0 (0–1)	0 (0–1)	0.624 ^a^
	Average of second cycle	0 (0–2)	0 (0–1)	0 (0–1)	0.262 ^a^
	Average of third cycle	0 (0–1)	0	0 (0–1)	0.019 ^a^*
	*p*-value	0.002 ^b^*	0.0157 ^b^*	0.0027 ^b^*	
NTX	At baseline	42 (40–43)	42 (41–43)	42 (41–43)	0.732 ^a^
	At the end of first cycle	40 (36–42)	41.5 (40–42.25)	41 (39–42)	0.041 ^a^*
	At the end of second cycle	39.5 (34.75–41)	41 (40–42)	41 (40–41)	0.007 ^a^*
	At the end of third cycle	39.5 (36–41)	41 (40–42)	41 (40–41)	0.002 ^a^*
	*p*-value	<0.0001 ^b^*	<0.0001 ^b^*	<0.0001 ^b^*	

^a^: Kruskal–Wallis test was used for statistical analysis. ^b^: Freidman ANOVA test was used for statistical analysis. *: Statistically significant, values are expressed as [median, interquartile range (IQR)], for all statistical tests used, *p*-value < 0.05 considered statistically significant. DN4: douleur neuropathique 4, NS: numeric pain rating scale, NTX: neurotoxicity subscale in the functional assessment of cancer therapy/gynecologic oncology group–neurotoxicity (FACT/GOG-Ntx).

**Table 4:** Comparison among the levels of selected biomarkers measured at baseline and after three cycles of vinca alkaloids.

Serum Biomarker	Assessment Time	Control (Group 1) *n* = 30	Diosmin/Hesperidin (Group 2) *n* = 30	Loratadine (Group 3) *n* = 30	*p*-Value
IL-1 β (pg/mL)	At baseline	16.75 (14.74–25.58)	21 (19.21–22.90)	20.85 (16.31–25.99)	0.9690 ^a^
	After treatment	36.80 (29.67–62.05)	26.06 (21.02–36.48)	26.18 (19.95–36.72)	0.0006 ^a^*
	*p*-value	<0.0001 ^b^*	0.0019 ^b^*	<0.0001 ^b^*	
TNF-α (ng/L)	At baseline	56.27 (54.54–57.39)	54.97 (51.84–59.20)	55.40 (46.29–62.05)	0.7915 ^a^
	After treatment	178.9 (142.2–196.5)	62.78 (58.06–74.85)	48.89 (43.48–64.02)	<0.0001 ^a^*
	Adjusted *p*-value	<0.0001 ^b^*	<0.0001 ^b^*	0.2534 ^b^*	
NGF (pg/mL)	At baseline	229.3 (211.7–239.6)	219.5 (192.6–284.6)	228.1 (177.7–283.1)	0.5400 ^a^
	After treatment	215.9 (182.9–235.5)	211.9 (193.3–244.1)	218.5 (175.1–285.5)	0.637 ^a^
	Adjusted *p*-value	0.0919 ^b^	0.6120 ^b^	0.5291 ^b^	
NFL (ng/L)	At baseline	22.17 (19.45–25.26)	21.25 (18.95–26.56)	21.09 (18.96–25.99)	0.9220 ^a^
	After treatment	153.3 (98.87–196.5)	45.73 (31.26–98.99)	37.09 (23.43–51.77)	<0.0001 ^a^*
	Adjusted *p*-value	<0.0001 ^b^*	<0.0001 ^b^*	<0.0001 ^b^*	

*: Statistically significant difference. ^a^: Comparisons were performed using the Kruskal–Wallis test. ^b^: Comparisons were performed utilizing the Wilcoxon matched-pairs signed rank test. Data are presented as [median, interquartile range (IQR)]. *p*-value < 0.05 is considered significant. IL1-β: interleukin 1-beta, TNF-α: tumor necrosis factor-alpha, NGF: nerve growth factor, NFL: neurofilament protein light polypeptide.

**Table 5:** The severity of neuropathy-related adverse effects in the three groups through the three cycles of vinca alkaloids.

Adverse Drug Effect	Assessment Time	Control (Group 1) *n* = 30	Diosmin/Hesperidin (Group 2) *n* = 30	Loratadine (Group 3) *n* = 30	*p*-Value
Constipation [*n*, (%)]	Through the first cycle	Grade 1: 10 (33.3%) Grade 2: 1 (3.3%) Grade 3: 1 (3.3%)	Grade 1: 11 (36.7%) Grade 2: 1 (3.3%) Grade 3: 0 (0%)	Grade 1: 9 (30%) Grade 2: 1 (3.3%) Grade 3: 0 (0%)	0.973 ^a^
	Through the second cycle	Grade 1: 9 (30%) Grade 2: 0 (0%) Grade 3: 1 (3.3%)	Grade 1: 8 (26.7%) Grade 2: 0 (0%) Grade 3: 0 (0%)	Grade 1: 5 (16.7%) Grade 2: 0 (0%) Grade 3: 0 (0%)	0.419 ^a^
	Through the third cycle	Grade 1: 9 (30%) Grade 2: 0 (0%) Grade 3:1 (3.3%)	Grade 1: 3 (10%) Grade 2: 0 (0%) Grade 3: 0 (0%)	Grade 1: 4 (13.3%) Grade 2: 0 (0%) Grade 3: 0 (0%)	0.094 ^a^
Paresthesia [*n*, (%)]	Through the first cycle	Grade 1: 5 (16.7%) Grade 2: 0 (0%)	Grade 1:2 (6.7%) Grade 2: 1 (3.3%)	Grade 1: 1 (3.3%) Grade 2: 0 (0%)	0.213 ^a^
	Through the second cycle	Grade 1: 6 (20%)	Grade 1: 0 (0%)	Grade 1: 1 (3.3%)	0.015 ^a^*
	Through the third cycle	Grade 1: 4 (13.3%)	Grade 1: 0 (0%)	Grade 1: 0 (0%)	0.032 ^a^*
Dysuria [*n*, (%)]	Through the first cycle	Grade 1: 4 (13.3%)	Grade 1: 2 (6.7%)	Grade 1: 2 (6.7%)	0.722 ^a^
	Through the second cycle	Grade 1: 1 (3.3%)	Grade 1: 0 (0%)	Grade 1: 0 (0%)	0.326 ^a^
	Through the third cycle	Grade 1: 4 (13.3%)	Grade 1: 1 (3.3%)	Grade 1: 0 (0%)	0.122 ^a^
Abdominal pain [*n*, (%)]	Through the first cycle	Grade 1: 5 (16.7%)	Grade 1: 2 (6.7%)	Grade 1: 4 (13.3%)	0.611 ^a^
	Through the second cycle	Grade 1: 2 (6.7%)	Grade 1: 4 (13.3%)	Grade 1: 6 (20%)	0.374 ^a^
	Through the third cycle	Grade 1: 1 (3.3%)	Grade 1: 3 (10%)	Grade 1: 2 (6.7%)	0.868 ^a^
Myalgia [*n*, (%)]	Through the first cycle	Grade 1: 2 (6.7%)	Grade 1: 0 (0%)	Grade 1: 1 (3.3%)	0.77 ^a^
	Through the second cycle	Grade 1: 2 (6.7%)	Grade 1: 0 (0%)	Grade 1: 0 (0%)	0.326 ^a^
	Through the third cycle	Grade 1: 0 (0%)	Grade 1: 0 (0%)	Grade 1: 1 (3.3%)	1 ^a^
Blurred vision [*n*, (%)]	Through the first cycle	Grade 1: 0 (0%)	Grade 1: 1 (3.3%)	Grade 1: 0 (0%)	0.326 ^a^
	Through the second cycle	Grade 1: 1 (3.3%)	Grade 1: 0 (0%)	Grade 1: 0 (0%)	1 ^a^
	Through the third cycle	Grade 1: 0 (0%)	Grade 1: 2 (6.7%)	Grade 1: 1 (3.3%)	0.318 ^a^

^a^: Fisher’s exact test was used for statistical analysis. *: Statistically significant. For all statistical tests used, a *p*-value < 0.05 is considered statistically significant.

## Data Availability

The datasets created and/or processed during the current trial are available upon reasonable request to the author for correspondence.

## Supplementary Materials

The following supporting information can be downloaded at: https://www.mdpi.com/article/10.3390/ph17050609/s1, Table S1. Results of kidney function tests among the three groups at baseline and at the end of every cycle of vinca alkaloids in the three groups. Table S2. Results of liver function tests among the three groups at baseline and at the end of every cycle of vinca alkaloids in the three groups. Table S3. Results of blood cells` counts among the three groups at baseline and at the end of every cycle of vinca alkaloids in the three groups in the three groups. Table S4. The functional assessment of cancer therapy/gynecologic oncology group –neurotoxicity (FACT/GOG-Ntx) score results in the three groups. Table S5. The severity of non-neuropathy related adverse effects in the three groups through the three cycles of Vinca alkaloids. Table S6. Common Terminology Criteria for Adverse Events (CTCAE) definitions for adverse effects encountered in the current study. Table S7. Mean adverse effect free time and hazard ratio of developing neuropathy related adverse drug effects among the three groups. Table S8. Noteworthy drug-drug interactions encountered during the study period. Figure S1. Numeric pain rating scale (NS) and douleur neuropathique 4 (DN4) pairwise comparisons at baseline and the end of every cycle of vinca alkaloids in each group. Figure S2. Physical well-being subscale scores in the three groups at baseline and at the end of every cycle of the three cycles of vinca alkaloids. Figure S3. Social well-being subscale scores in the three groups at baseline and at the end of every cycle of the three cycles of vinca alkaloids. Figure S4. Emotional well-being subscale scores in the three groups at baseline and at the end of every cycle of the three cycles of vinca alkaloids. Figure S5. Functional well-being subscale scores in the three groups at baseline and at the end of every cycle of the three cycles of vinca alkaloids. Figure S6. Neurotoxicity subscale scores in the three groups at baseline and at the end of every cycle of the three cycles of vinca alkaloids. Figure S7. FACT/GOG NTx total scores in the three groups at baseline and at the end of every cycle of the three cycles of vinca alkaloids. Figure S8. Cumulative survival comparisons between the three groups with constipation, paresthesia, dysuria, abdominal pain, myalgia, and blurred vision as the event of interest. Figure S9. Cumulative hazard comparisons between the three groups with constipation, paresthesia, dysuria, abdominal pain, myalgia, and blurred vision as the event of interest. Figure S10. Considerable correlations of neuropathy scores and serum biomarkers.
