# Supplementary material for: Evaluation of the Effect of Loratadine versus Diosmin/Hesperidin Combination on Vinca Alkaloids-Induced Neuropathy: A Randomized Controlled Clinical Trial
Source: Pharmaceuticals (Basel). 2024 May 9;17(5):609. doi: 10.3390/ph17050609 (PMC11124025; doi:10.3390/ph17050609)
Supplement: Supplementary file 1 [file pharmaceuticals-17-00609-s001.zip › pharmaceuticals-2979506-supplementary.pdf]

Supplementary Materials

Evaluation of The Effect of Loratadine versus Diosmin/Hesperidin Combination on Vinca Alkaloids-Induced Neuropathy: A Randomized Controlled Clinical Trial

Noha Kamal [1,*], Mahmoud S. Abdallah [1,2], Essam Abdel Wahed [3], Nagwa A. Sabri [4] and Sarah Farid Fahmy [4]

[1] Clinical Pharmacy Department, Faculty of Pharmacy, University of Sadat City (USC), Sadat City 32897, Egypt
[2] Department of PharmD, Faculty of Pharmacy, Jadara University, Irbid 21110, Jordan
[3] Hematology and Bone Marrow Transplantation Unit, Internal Medicine Department, Faculty of Medicine, Ain Shams University, Cairo 11591, Egypt
[4] Clinical Pharmacy Department, Faculty of Pharmacy, Ain Shams University, African Union Organization Street, Cairo 11566, Egypt; nagwa.sabri@pharma.asu.edu.eg (N.A.S.); sarah.farid@pharma.asu.edu.eg (S.F.F.)
* Correspondence: noha.kamal@fop.usc.edu.eg; Tel.: +20-1002014552

Table S1. Results of kidney function tests among the three groups at baseline and at the end of every cycle of vinca alkaloids in the three groups.

Parameters		Group 1 Control (n=30)	Group 2 Diosmin/ Hesperidin (n=30)	Group 3 Loratadine (n=30)	*p*-values
**Serum creatinine (mg/dL)**	At baseline	0.79 (0.6-0.925)	0.8 (0.6-0.9)	0.8 (0.7-1)	0.324[a]
	At the end of 1st cycle	0.8 (0.6-0.9)	0.7 (0.6-0.8)	0.7 (0.6-0.9)	0.527[a]
	At the end of 2nd cycle	0.7 (0.5-0.925)	0.7 (0.6-0.8)	0.7 (0.6-0.825)	0.981[a]
	At the end of 3rd cycle	0.7 (0.5-0.87)	0.7 (0.58-0.9)	0.7 (0.6-0.9)	0.962[a]
	***p*-value**	0.380[b]	0.645[b]	<0.001[b]*	
**BUN (mg/dL)**	At baseline	14 (11-17.25)	13 (10.5-18)	14 (11.75-18)	0.753[a]
	At the end of 1st cycle	15.5 (12-22)	17 (11.75-21)	19.5 (15-22.5)	0.152[a]
	At the end of 2nd cycle	16 (12.75-21)	16 (11-20)	20.5 (15.3-23.3)	0.089[a]
	At the end of 3rd cycle	15.5 (12-22.25)	16 (10.75-22.5)	17 (13-24)	0.435[a]
	***p*-value**	0.046[b]*	0.138[b]	0.004[b]*	
**eGFR (ml/min/1.7m²)**	At baseline	106 (97.5-128.5)	114 (90-124.8)	97 (86.75-120)	0.198[a]
	At the end of 1st cycle	114.5 (90.75-131)	119.5 (105.8-132.3)	106.5 (95.5-125)	0.491[a]
	At the end of 2nd cycle	116 (90-132)	113.5 (106.5-131.3)	110 (96.75-127.3)	0.741[a]
	At the end of 3rd cycle	116 (95.75-137)	121 (103.8-133.3)	109.5 (99.5-124)	0.561[a]
	***p*-value**	0.266[b]	0.723[b]	0.0002[b]*	

[a]: Kruskal Wallis test was used for statistical analysis, [b]: Freidman ANOVA test was used for statistical analysis, *: statistically significant, values are expressed as [median, (IQR)], for all statistical tests used $p<0.05$ considered statistically significant. BUN: Blood Urea Nitrogen, eGFR: estimated glomerular filtration rate calculated utilizing chronic kidney disease epidemiology collaboration (CKD-EPI) equation

Table S2. Results of liver function tests among the three groups at baseline and at the end of every cycle of vinca alkaloids in the three groups.

	Parameters	Group 1 Control (n=30)	Group 2 Diosmin/ Hesperidin (n=30)	Group 3 Loratadine (n=30)	*p*-values
Direct bilirubin	At baseline	0.25 (0.1- 0.425)	0.2 (0.1-0.3)	0.16 (0.1-0.225)	0.0586[a]
	At the end of 1st cycle	0.2 (0.175-0.5)	0.2 (0.1-0.3)	0.2 (0.1-0.2)	0.0148[a]*
	At the end of 2nd cycle	0.2 (0.1-0.48)	0.2 (0.1-0.3)	0.2 (0.1-0.3)	0.5661[a]
	At the end of 3rd cycle	0.2 (0.1-0.325)	0.2 (0.1-0.225)	0.2 (0.1-0.3)	0.2616[a]
	***p*-value**	0.901[b]	0.886[b]	0.143[b]	
Total bilirubin	At baseline	0.7 (0.55-1.025)	0.7 (0.475-1)	0.7 (0.575-0.825)	0.8729[a]
	At the end of 1st cycle	0.5 (0.3-0.75)	0.65 (0.40.8)	0.6 (0.475-0.7)	0.499[a]
	At the end of 2nd cycle	0.65 (0.3-0.875)	0.55 (0.4-0.925)	0.7 (0.575-0.8)	0.597[a]
	At the end of 3rd cycle	0.6 (0.4- 0.825)	0.55 (0.3-0.725)	0.7 (0.55-0.925)	0.123[a]
	***p*-value**	0.280[b]	.012[b]*	0.051[b]	
AST	At baseline	25.5 (18-38.5)	23.5 (16.75-29.75)	29.5 (22-36)	0.3701[a]
	At the end of 1st cycle	30 (15.75-47)	17.5 (13.75-26)	28 (16-37)	0.039[a]
	At the end of 2nd cycle	28 (16-44.25)	21 (14.75-32.25)	30.5 (17.25-44)	0.137[a]
	At the end of 3rd cycle	22.5 (14.75- 31.5)	19.5 (14.75-29.75)	28 (18.75- 43.25)	0.035[a]*
	***p*-value**	0.065[b]	0.561[b]	0.441[b]	
ALT	At baseline	26.5 (16-36.5)	18.5 (11.75-24.75)	19 (15-34)	0.260[a]
	At the end of 1st cycle	26.5 (11-44.25)	16.5 (11-31.25)	21.5 (12.75-34.25)	0.385[a]
	At the end of 2nd cycle	22 (15.75-43.5)	18 (14.75-28)	24 (16.75-40.5)	0.140[a]
	At the end of 3rd cycle	19 (12.75-36.25)	17.5 (10.75-25)	29.5 (18- 47)	0.014[a]*
	***p*-value**	0 .756[b]	0.824[b]	0.064[b]	

[a]: Kruskal Wallis test was used for statistical analysis, [b]: Freidman ANOVA test was used for statistical analysis, *: statistically significant, values are expressed as [median, (IQR)], for all statistical tests used $p<0.05$ considered statistically significant. ALT: Alanine Aminotransferase, AST: Aspartate Aminotransferase.

Table S3. Results of blood cells` counts among the three groups at baseline and at the end of every cycle of vinca alkaloids in the three groups in the three groups.

	Parameters	Group 1<br>Control<br>(n=30)	Group 2<br>Diosmin/ Hesperidin<br>(n=30)	Group 3<br>Loratadine<br>(n=30)	*p*-values
**Hemoglobin**	At baseline	8.7 (7.58-10.08)	8.6 (7.05-9.78)	8.45 (7.28-10.25)	0.825[a]
	At the end of 1st cycle	9.05 (8.6-10.1)	9.5 (8.83-10)	8.45 (8.15-10.625)	0.371[a]
	At the end of 2nd cycle	9.25 (8.5-10.38)	9.45 (9-10.5)	9.3 (8.73-9.85)	0.455[a]
	At the end of 3rd cycle	10 (9.35-10.73)	9.7 (8.85-10.45)	9.3 (8.73-9.85)	0.105[a]
	***p*-value**	0.038[b]*	0.058[b]	0.27[b]	
**Platelets**	At baseline	77.5 (39.5-145.5)	71.5 (25.75-111.8)	50.5 (25.25-201.5)	0.708[a]
	At the end of 1st cycle	90 (63.5-201.75)	112 (74-175)	100.5 (38.98 -203)	0.927[a]
	At the end of 2nd cycle	111.5 (66.3 -246.3)	144.5 (90.75-180.5)	120 (50.75-175.5)	0.469[a]
	At the end of 3rd cycle	132.5 (96.5-235)	141.5 (103.5-237)	142.5 (50-195)	0.485[a]
	***p*-value**	0.046[b]*	<0.001[b]*	0.265[b]	
**Neutrophils**	At baseline	2.56 (1.46-5.4)	2.2 (0.58-6.7825)	2.995(0.98-5.38)	0.942[a]
	At the end of 1st cycle	2.12 (1.075-4.38)	2.55 (1.65-5.84)	3 (1.36-5.85)	0.811[a]
	At the end of 2nd cycle	2.24 (1.5- 4.85)	2.2 (1.78-5.28)	2.45 (1.98-4.125)	0.807[a]
	At the end of 3rd cycle	2.82 (1.7-5.53)	2.35 (2.07-4.55)	2.85 (2.09-4.725)	0.950[a]
	***p*-value**	0.289[b]	0.581[b]	0.716[b]	
**TLC**	At baseline	5.75 (2.898-35.25)	6.4 (3-35.4)	9.05 (2.925-53.05)	0.530[a]
	At the end of 1st cycle	5.1 (2.6-8.13)	4.95 (3.15-11.58)	7.25 (4.25-14.55)	0.160[a]
	At the end of 2nd cycle	5.95 (3.1-7.68)	5.05 (3.33-10.25)	6.25 (3.98-12.25)	0.5[a]
	At the end of 3rd cycle	6.2 (3.65-9.1)	6.2 (4.55-11.59)	7 (4.2-11.3)	0.632[a]
	***p*-value**	0.247[b]	0.101[b]	0.233[b]	

[a]: Kruskal Wallis test was used for statistical analysis, [b]: Freidman ANOVA test was used for statistical analysis, *: statistically significant, values are expressed as [median, (IQR)], for all statistical tests used $p<0.05$ considered statistically significant. TLC: Total Leucocyte Count. Within group1, the hemoglobin level increased across the cycles but there was no significant difference compared to other groups. In group 2, platelets count increased from baseline to the 2nd cycle (p<0.001) and 3rd cycle (p<0.001). Platelets counts also increased in group 1 after the end of 3rd cycle (p=0.031) compared to baseline in group 1. The platelet count increased in group 3 as well, although it was not deemed significantly different.

Table S4. The functional assessment of cancer therapy/gynecologic oncology group–neurotoxicity (FACT/GOG-Ntx) score results in the three groups.

Subscale		Group 1 Control (n=30)	Group 2 Diosmin/Hesperidin (n=30)	Group 3 Loratadine (n=30)	*p*-values
PWB	At baseline	19.75 (16-22.5)	22.5 (17.75-24)	21 (17.75-25)	0.737[a]
	At the end of 1st cycle	17 (16-20)	21 (15.75-23)	19 (16-23)	0.950[a]
	At the end of 2nd cycle	16 (14-17)	19.5 (14.75-21)	17.5 (14.75-22)	0.748[a]
	At the end of 3rd cycle	14.75 (12-16)	18.5 (13.75-20)	16 (13-20.25)	0.594[a]
	***p*-value**	<0.0001[b]*	<0.0001[b]*	<0.0001[b]*	
SWB	At baseline	21 (18-23)	21 (17.75-24)	23.5 (19-25)	0.206[a]
	At the end of 1st cycle	21 (17.75-22)	20 (17-23.25)	22.5 (19-24.25)	0.317[a]
	At the end of 2nd cycle	20 (15.75-22)	20 (16.75-22)	21 (18-23.25)	0.5[a]
	At the end of 3rd cycle	20 (16-22)	19.5 (17-22)	20 (18-23.25)	0.503[a]
	***p*-value**	<0.0001[b]*	<0.0001[b]*	<0.0001[b]*	
EWB	At baseline	20 (18.75-22)	21 (18-22)	20 (19-22)	0.764[a]
	At the end of 1st cycle	19 (17.75- 21)	20 (18-21.25)	20 (18.75-21)	0.719[a]
	At the end of 2nd cycle	18 (17-19)	19.5 (17-21)	19 (17-20)	0.234[a]
	At the end of 3rd cycle	17 (16-18.25)	19 (16.75-20)	18 (16-19)	0.186[a]
	***p*-value**	<0.0001[b]*	<0.0001[b]*	<0.0001[b]*	
FWB	At baseline	17 (13.75-19)	16 (13.75-18)	16.5 (13.75-19)	0.945[a]
	At the end of 1st cycle	15 (13.75-18)	15 (12.75-17)	15 (12.75-17)	0.802[a]
	At the end of 2nd cycle	14 (12-18)	14 (12-16)	14 (11-16)	0.5[a]
	At the end of 3rd cycle	13 (11-17)	13.5 (11-15.25)	13.5 (10-15)	0.514[a]
	***p*-value**	<0.0001[b]*	<0.0001[b]*	<0.0001[b]*	

Cont. Table S4. The functional assessment of cancer therapy/gynecologic oncology group–neurotoxicity (FACT/GOG-Ntx) score results in the three groups.

**FACT/GOG-Ntx**	At baseline	120 (115-125.3)	120.5 (113.3-125.5)	123 (116-127)	0.932[a]
	At the end of 1st cycle	114 (108.8-117.3)	116 (108-122.5)	115 (111-124)	0.655[a]
	At the end of 2nd cycle	106 (104-112.8)	112 (104.5-118.3)	109 (105-117)	0.16[a]
	At the end of 3rd cycle	104 (100.5-108)	110.5 (101.8-116.3)	107 (101-115)	0.217[a]
	***p*-value**	<0.0001[b]*	<0.0001[b]*	<0.0001[b]*	

[a]: Kruskal Wallis test was used for statistical analysis, [b]: Freidman ANOVA test was used for statistical analysis, N.B. the table excludes neuropathy subscale as it is presented in the original paper, *: statistically significant, values are expressed as [median, (IQR)], for all statistical tests used $p<0.05$ considered statistically significant. EWB: emotional well-being, FACT/GOG-Ntx: functional assessment of cancer therapy/gynecologic oncology group –neurotoxicity total score, FWB: Functional Well-Being, PWB: physical well-being, and SWB: social/family well-being

Table S5. The severity of non-neuropathy related adverse effects in the three groups through the three cycles of vinca alkaloids.

Adverse drug effect		Group 1 (n=30)	Group 2 (n=30)	Group 3 (n=30)	*p*-values
**Bone Pain [n, (%)]**	Through the 1st cycle	Grade 1: 1 (3.3%)	Grade 1: 1 (3.3%)	Grade 1: 3 (10%)	0.613[a]
	Through the 2nd cycle	Grade 1: 1 (3.3%)	Grade 1: 0 (0%)	Grade 1: 2 (6.7%)	0.77[a]
	Through the 3rd cycle	Grade 1: 0 (0%)	Grade 1: 0 (0%)	Grade 1: 1 (3.3%)	0.521[a]
**Headache [n, (%)]**	Through the 1st cycle	Grade 1: 3 (10%)	Grade 1: 0 (0%)	Grade 1: 0 (0%)	0.149[a]
	Through the 2nd cycle	Grade 1: 0 (0%)	Grade 1: 1 (3.3%)	Grade 1: 1 (3.3%)	1[a]
	Through the 3rd cycle	Grade 1: 0 (0%)	Grade 1: 1 (3.3%)	Grade 1: 0 (0%)	1[a]
**Nausea [n, (%)]**	Through the 1st cycle	Grade 1: 1 (3.3%)	Grade 1: 0 (0%)	Grade 1: 1 (3.3%)	1[a]
	Through the 2nd cycle	Grade 1: 0 (0%)	Grade 1: 1 (3.3%)	Grade 1: 3 (10%)	0.122[a]
		Grade 2: 0 (0%)	Grade 2: 0 (0%)	Grade 2: 1 (3.3%)	
	Through the 3rd cycle	Grade 1: 1 (3.3%)	Grade 1: 0 (0%)	Grade 1: 0 (0%)	1[a]
**Vomiting [n, (%)]**	Through the 1st cycle	Grade 1: 4 (13.3%)	Grade 1: 3 (10%)	Grade 1: 8 (26.7%)	0.326[a]
		Grade 2: 4 (13.3%)	Grade 2: 3 (10%)	Grade 2: 1 (3.3%)	
	Through the 2nd cycle	Grade 1: 2 (6.7%)	Grade 1: 4 (13.3%)	Grade 1: 3 (10%)	0.584[a]
		Grade 2: 1 (3.3%)	Grade 2: 2 (6.7%)	Grade 2: 0 (0%)	
	Through the 3rd cycle	Grade 1: 4 (13.3%)	Grade 1: 1 (3.3%)	Grade 1: 4 (13.3%)	0.204[a]
		Grade 2: 2 (6.7%)	Grade 2: 0 (0%)	Grade 2: 0 (0%)	
**Bilirubin increased [n, (%)]**	Through the 1st cycle	Grade 3: 1 (3.3%)	Grade 3: 0 (0%)	Grade 3: 0 (0%)	1[a]
	Through the 2nd cycle	Grade 1: 2 (6.7%)	Grade 1: 2 (6.7%)	Grade 1: 0 (0%)	0.540[a]
		Grade 3: 0 (0%)	Grade 3: 0 (0%)	Grade 3: 1 (3.3%)	
	Through the 3rd cycle	Grade 2: 0 (0%)	Grade 2: 0 (0%)	Grade 2: 1 (3.3%)	1[a]
**ALT increased [n, (%)**	Through the 1st cycle	Grade 1: 1 (3.3%)	Grade 1: 0 (0%)	Grade 1: 1 (3.3%)	1[a]
	Through the 2nd cycle	Grade 1: 3 (10%)	Grade 1: 1 (3.3%)	Grade 1: 0 (0%)	0.318[a]
		Grade 3: 0 (0%)	Grade 3: 0 (0%)	Grade 3: 1 (3.3%)	
	Through the 3rd cycle	Grade 1: 0 (0%)	Grade 1: 0 (0%)	Grade 1: 2 (6.7%)	0.326[a]

Cont. Table S5. The severity of non-neuropathy related adverse effects in the three groups thvrough the three cycles of vinca alkaloids.

**AST increased [n, (%)]**	Through the 1st cycle	Grade 1: 1 (3.3%)	Grade 1: 0 (0%)	Grade 1: 1 (3.3%)	1[a]
	Through the 2nd cycle	Grade 1: 3 (10%)<br>Grade 3: 0 (0%)	Grade 1: 1 (3.3%)<br>Grade 3: 0 (0%)	Grade 1: 0 (0%)<br>Grade 3: 1 (3.3%)	0.318[a]
	Through the 3rd cycle	Grade 1: 0 (0%)	Grade 1: 0 (0%)	Grade 1: 2 (6.7%)	0.326[a]
**Diarrhea [n, (%)]**	Through the 1st cycle	Grade 1: 2 (6.7%)	Grade 1: 5 (16.7%)	Grade 1: 3 (10%)	0.592[a]
	Through the 2nd cycle	Grade 1: 1 (3.3%)	Grade 1: 0 (0%)	Grade 1: 0 (0%)	1[a]
	Through the 3rd cycle	Grade 1: 0 (0%)	Grade 1: 3 (10%)	Grade 1: 1 (3.3%)	0.318[a]
**Edema [n, (%)]**	Through the 1st cycle	Grade 1 Edema limbs: 2 (6.7%)	Grade 1 Edema limbs: 2 (6.7%)	Grade 1 Edema limbs: 2 (6.7%)	1[a]
	Through the 2nd cycle	Grade 1 Edema limbs: 2 (6.7%)<br>Grade 1 Edema Face: 0 (0%)	Grade 1 Edema limbs: 2 (6.7%)<br>Grade 1 Edema Face: 1 (3.3%)	Grade 1 Edema limbs: 0 (0%)<br>Grade 1 Edema Face: 0 (0%)	0.463[a]
	Through the 3rd cycle	Grade 1 Edema limbs: 1 (3.3%)<br>Grade 1 Edema Face: 0 (0%)	Grade 1 Edema limbs: 1 (3.3%)<br>Grade 1 Edema Face: 1 (3.3%)	Grade 1 Edema limbs: 0 (0%)<br>Grade 1 Edema Face: 0 (0%)	0.77[a]

[a]: Fisher's Exact Test was used for statistical analysis. AST: Aspartate Aminotransferase, ALT: Alanine Aminotransferase. For all statistical tests used, $p<0.05$ is considered statistically significant.

Table S6. Common Terminology Criteria for Adverse Events (CTCAE) definitions for adverse effects encountered in the current study.

**Abdominal pain**	
Definition	"A disorder characterized by a sensation of marked discomfort in the abdominal region."
Grades	Grade 1: "Mild pain."
**Alanine aminotransferase increased**	
Definition	"A finding based on laboratory test results that indicate an increase in the level of alanine aminotransferase (ALT or SGPT) in the blood specimen."
Grades	Grade 1: ">ULN - 3.0 x ULN if baseline was normal; 1.5 - 3.0 x baseline if baseline was abnormal."
**Aspartate aminotransferase increased**	
Definition	"A finding based on laboratory test results that indicate an increase in the level of aspartate aminotransferase (AST or SGOT) in a blood specimen."
Grades	Grade 1: ">ULN - 3.0 x ULN if baseline was normal; 1.5 - 3.0 x baseline if baseline was abnormal."
**Blood bilirubin increased**	
Definition	"A finding based on laboratory test results that indicate an abnormally high level of bilirubin in the blood. Excess bilirubin is associated with jaundice."
Grades	Grade 1: ">ULN - 1.5 x ULN if baseline was normal; > 1.0 - 1.5 x baseline if baseline was abnormal."
**Blurred vision**	
Definition	"A disorder characterized by visual perception of unclear or fuzzy images."
Grades	Grade 1: "Intervention not indicated."
**Bone pain**	
Definition	"A disorder characterized by a sensation of marked discomfort in the bones."
Grades	Grade 1: "Mild pain."

Cont. Table S6. Common Terminology Criteria for Adverse Events (CTCAE) definitions for adverse effects encountered in the current study.

**Constipation**	
Definition	"A disorder characterized by irregular and infrequent or difficult evacuation of the bowels."
Grades	Grade 1: "Occasional or intermittent symptoms; occasional use of stool softeners, laxatives, dietary modification, or enema."
**Diarrhea**	
Definition	"A disorder characterized by an increase in frequency and/or loose or watery bowel movements."
Grades	Grade 1: "Increase of <4 stools per day over baseline; mild increase in ostomy output compared to baseline."
**Dysuria**	
Definition	"A disorder characterized by painful urination."
Grades	Grade 1: "Present."
**Edema face**	
Definition	"A disorder characterized by swelling due to excessive fluid accumulation in facial tissues."
Grades	Grade 1: "Localized facial edema."
**Edema limbs**	
Definition	"A disorder characterized by swelling due to excessive fluid accumulation in the upper or lower extremities."
Grades	Grade 1: "5 - 10% inter-limb discrepancy in volume or circumference at point of greatest visible difference; swelling or obscuration of anatomic architecture on close inspection."

Cont. Table S6. Common Terminology Criteria for Adverse Events (CTCAE) definitions for adverse effects encountered in the current study.

**Headache**	
Definition	"A disorder characterized by a sensation of marked discomfort in various parts of the head, not confined to the area of distribution of any nerve."
Grades	Grade 1: "Mild pain."
**Myalgia**	
Definition	"A disorder characterized by marked discomfort sensation originating from a muscle or group of muscles."
Grades	Grade 1: "Mild pain."
**Nausea**	
Definition	"A disorder characterized by a queasy sensation and/or the urge to vomit."
Grades	Grade 1: "Loss of appetite without alteration in eating habits."
**Paresthesia**	
Definition	"A disorder characterized by functional disturbances of sensory neurons resulting in abnormal cutaneous sensations of tingling, numbness, pressure, cold, and/or warmth.".
Grades	Grade 1: "Mild symptoms."
**Vomiting**	
Definition	"A disorder characterized by the reflexive act of ejecting the contents of the stomach through the mouth."
Grades	Grade 1: "Intervention not indicated."

Table S7. Mean adverse effect free time and hazard ratio of developing neuropathy related adverse drug effects among the three groups.

Neuropathy related adverse effect	Group 1 Mean adverse effect free time (months)	Group 2 Mean adverse effect free time (months)	Group 3 Mean adverse effect free time (months)	Hazard ratio	95% Confidence interval of hazard ratio
Constipation	2.58	2.61	2.7	0.766	(0.577-1.018)
Paresthesia	2.79	2.93	2.96	0.304	(0.146-0.631)
Dysuria	2.89	2.95	2.95	0.434	(0.204-0.932)
Abdominal pain	2.86	2.89	2.81	1.232	(0.758-1.933)
Myalgia	2.92	2.97	3	0.347	(0.087-1.377)
Blurred vision	2.98	2.97	3	0.974	(0.331-2.863)

Mean adverse effect free time was computed using Kaplan Meier analysis, Cox regression analysis was utilized for calculation of hazard ratios with group 3 as the reference group and 95% confidence intervals, adverse effect free time is expressed in months.

Table S8. Noteworthy drug-drug interactions encountered during the study period.

Study group (number of patients)	Interacting medications	Risk rating	Mechanism	Recommendation
**Group 1: (2)**<br>**Group 2: (3)**<br>**Group 3: (2)**	**Amphotericin B and Prednisolone**	**C**	Corticosteroids (Systemic) may enhance the hypokalemic effect of Amphotericin B.	Monitor cardiac function and serum electrolytes (especially potassium) if systemic corticosteroids are co-administered with amphotericin B.
**Group 1: (3)**<br>**Group 2: (5)**<br>**Group 3: (4)**	**Amphotericin B and Vinca alkaloids/Cyclophosphamide/Doxorubicin**	**C**	Antineoplastic Agents may enhance the adverse/toxic effect of Amphotericin B.	Monitor for possible increases in renal toxicity, bronchospasm, and hypotension if amphotericin is given concomitantly with antineoplastic agents.
**Group 1: (2)**<br>**Group 2: (4)**<br>**Group 3: (0)**	**Cyclophosphamide and Doxorubicin**	**C**	Cyclophosphamide may enhance the cardiotoxic effect of doxorubicin.	Monitor cardiac function closely. The cardiotoxic effects of these agents may be additive or synergistic. Administration of cyclophosphamide by infusion or twice daily or using liposomal anthracycline formulations may reduce risk.
**Group 1: (0)**<br>**Group 2 :(2)**<br>**Group 3:(1)**	**Cyclophosphamide and Hydrochlorothiazide**	**C**	Thiazide Diuretics may enhance the adverse/toxic effect of cyclophosphamide. Specifically, granulocytopenia may be enhanced.	Monitor for signs and symptoms of hematological toxicity.

Cont. Table S8. Noteworthy drug-drug interactions encountered during the study period.

Study group (number of patients)	Interacting medications	Risk rating	Mechanism	Recommendation
**Group 1: (0) Group 2: (26) Group 3: (0)**	**Diosmin and doxorubicin***	**X***	P-glycoprotein/ABCB1 Inhibitors may increase the serum concentration of doxorubicin (Conventional).	Avoid concomitant use of doxorubicin and P-glycoprotein (P-gp) inhibitors.
**Group 1: (1) Group 2: (2) Group 3: (1)**	**Fluconazole and ciprofloxacin**	**C**	Corticosteroids (Systemic) may enhance the adverse/toxic effect of Quinolones.	Monitor patients closely for new-onset tendon or joint pain. The risk may be further increased in older patients (> 60 years) and in recipients of heart, lung, and kidney transplants.
**Group 1: (1) Group 2: (1) Group 3: (2)**	**Fluconazole and doxorubicin**	**X**	Moderate CYP3A4 Inhibitors (Fluconazole) may increase the serum concentration of doxorubicin (Conventional).	Avoid coadministration of doxorubicin with moderate CYP3A4 inhibitors due to the risk of increased doxorubicin adverse effects.

Cont. Table S8. Noteworthy drug-drug interactions encountered during the study period.

Study group (number of patients)	Interacting medications	Risk rating	Mechanism	Recommendation
**Group 1: (22)**<br>**Group 2: (20)**<br>**Group 3: (23)**	**Fluconazole and levofloxacin**	**C**	QT-prolonging Quinolone Antibiotics (Levofloxacin) may enhance the QTc-prolonging effect of QT-prolonging Moderate CYP3A4 Inhibitors (Fluconazole).	Monitor for QTc interval prolongation and ventricular arrhythmias. Patients with other risk factors (eg, older age, female sex, bradycardia, hypokalemia, hypomagnesemia, heart disease, and higher drug concentrations) are likely at greater risk for these potentially life-threatening toxicities.
**Group 1: (4)**<br>**Group 2: (3)**<br>**Group 3: (5)**	**Fluconazole and ondansetron**	**C**	Ondansetron may enhance the QTc-prolonging effect of QT-prolonging Moderate CYP3A4 Inhibitors (ondansetron)	
**Group 1: (25)**<br>**Group 2: (26)**<br>**Group 3: (25)**	**Fluconazole and vincristine**	**C**	Fluconazole may increase the serum concentration of vincristine.	Monitor for increased vincristine toxicities.
**Group 1: (0)**<br>**Group 2: (1)**<br>**Group 3: (1)**	**Hydrochlorothiazide and prednisolone**	**C**	Corticosteroids (Systemic) may enhance the hypokalemic effect of Thiazide and Thiazide-Like Diuretics.	Monitor serum potassium. The addition of potassium-sparing diuretic and/or potassium supplementation may be necessary with concomitant treatment.
**Group 1: (0)**<br>**Group 2: (0)**<br>**Group 3: (1)**	**Ketoprofen and prednisolone**	**C**	Corticosteroids (Systemic) may enhance the adverse/toxic effect of Nonsteroidal Anti-Inflammatory Agents (Nonselective)	Monitor for signs of bleeding. Concomitant use may increase the risk of gastrointestinal bleeding.

Cont. Table S8. Noteworthy drug-drug interactions encountered during the study period.

**Group 1: (0)** **Group 2: (0)** **Group 3: (1)**	**Loratadine and hydrochlorothiazide**	**C**	Anticholinergic Agents (Loratadine) may increase the serum concentration of Thiazide and Thiazide-Like Diuretics.	Monitor for an increased response to thiazide diuretics during concomitant treatment with an anticholinergic agent, particularly when anticholinergic doses are sufficient to reduce gastrointestinal motility.
**Group 1: (1)** **Group 2: (0)** **Group 3: (0)**	**Rifampicin and vincristine**	**C**	Strong CYP3A4 Inducers (Rifampicin) may decrease the serum concentration of vincristine.	Monitor for reduced vincristine efficacy if combined with rifampicin.
**Group 1: (1)** **Group 2: (2)** **Group 3: (2)**	**Voriconazole and prednisolone**	**C**	Strong CYP3A4 Inhibitors (voriconazole) may increase the serum concentration of prednisolone.	Monitor for increased steroid-related adverse effects.
**Group 1: (1)** **Group 2: (2)** **Group 3: (3)**	**Voriconazole and Vincristine**	**D**	Strong CYP3A4 Inhibitors (voriconazole) may increase the serum concentration of vincristine.	Seek alternatives to this combination when possible. If combined, monitor closely for vincristine toxicities.

C: Monitor therapy, D: Consider therapy modification, X: Avoid combination. All drug-drug interactions are produced utilizing online Lexicomp® interaction checker last accessed 25th May 2023. *: The interaction recommendation is based on clinical trials of zosuquidar trihydrochloride and cyclosporin interacting with doxorubicin [1,2]. However, diosmin has been evaluated in several studies in which it reduced doxorubicin adverse effects such as nephrotoxicity [3], cardiotoxicity [4] and hepatotoxicity [5,6] and enhanced its antitumor activity[7]

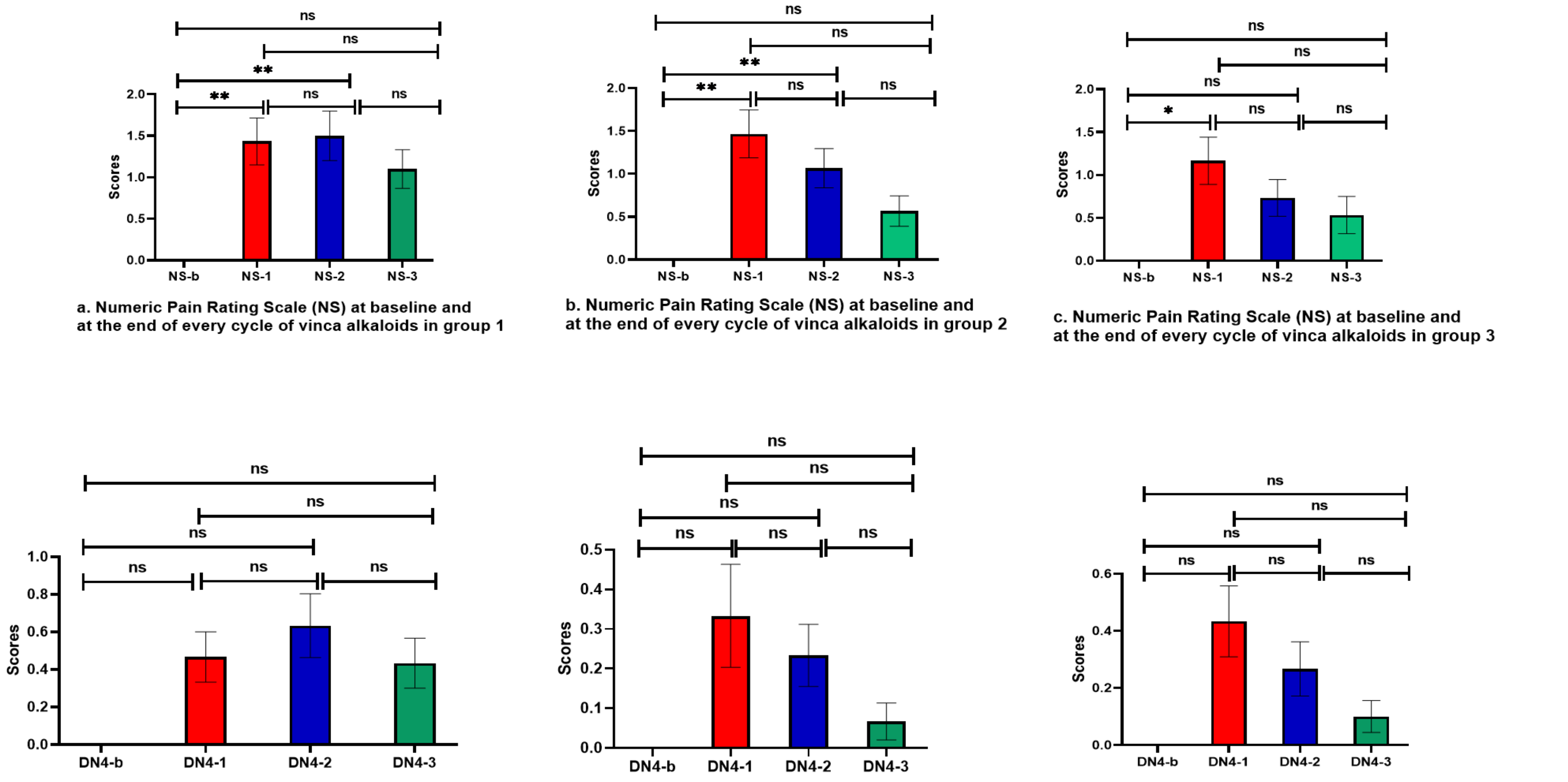

a. Numeric Pain Rating Scale (NS) at baseline and at the end of every cycle of vinca alkaloids in group 1

b. Numeric Pain Rating Scale (NS) at baseline and at the end of every cycle of vinca alkaloids in group 2

c. Numeric Pain Rating Scale (NS) at baseline and at the end of every cycle of vinca alkaloids in group 3

e. Neuropathic Pain in 4 questions (DN4) at baseline and at the end of every cycle of vinca alkaloids in group 1

f. Neuropathic Pain in 4 questions (DN4) at baseline and at the end of every cycle of vinca alkaloids in group 2

g. Neuropathic Pain in 4 questions (DN4) at baseline and at the end of every cycle of vinca alkaloids in group 3

Figure S1. Numeric pain rating scale (NS) and douleur neuropathique 4 (DN4) pairwise comparisons at baseline and the end of every cycle of vinca alkaloids in each group.

Pairwise comparisons were done utilizing Dunn's correction, data are expressed as median with range, ns: not statistically significant, ✳: p<0.05, ✳✳:p<0.01, ✳✳✳:p<0.001.

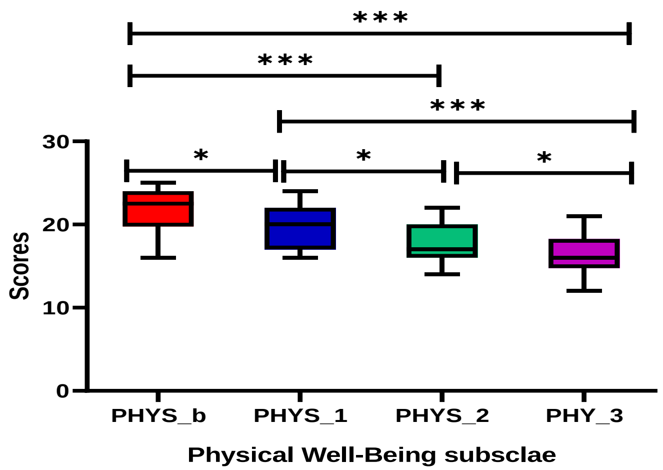

a. Physical Well-Being Subscale scores in group 1 at baseline and at the end of every cycle of Vinca alkaloids

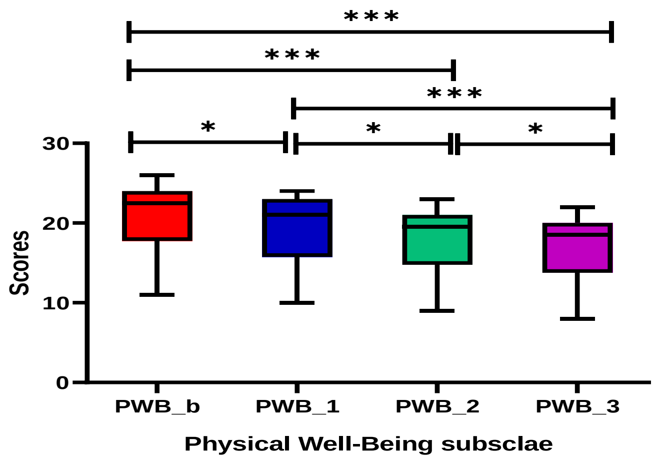

b. Physical Well-Being Subscale scores in group 2 at baseline and at the end of every cycle of Vinca alkaloids

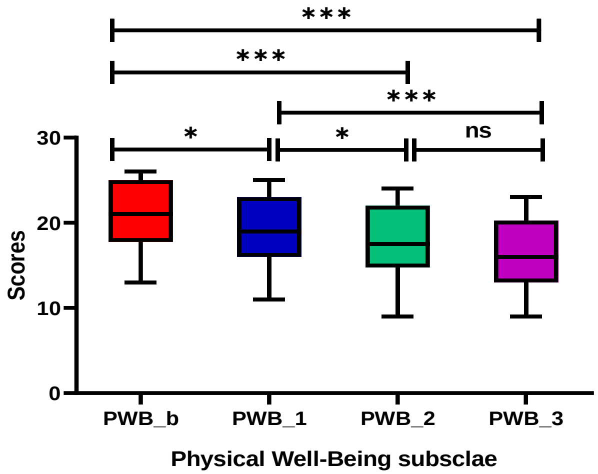

c. Physical Well-Being Subscale scores in group 3 at baseline and at the end of every cycle of Vinca alkaloids

Figure S2. Physical well-being subscale scores in the three groups at baseline and at the end of every cycle of the three cycles of vinca alkaloids.

Pairwise comparisons were done utilizing Dunn`s correction, data are expressed as median and IQR, ns: not statistically significant, ✱: $p<0.05$, ✱✱: $p<0.01$, ✱✱✱: $p<0.001$.

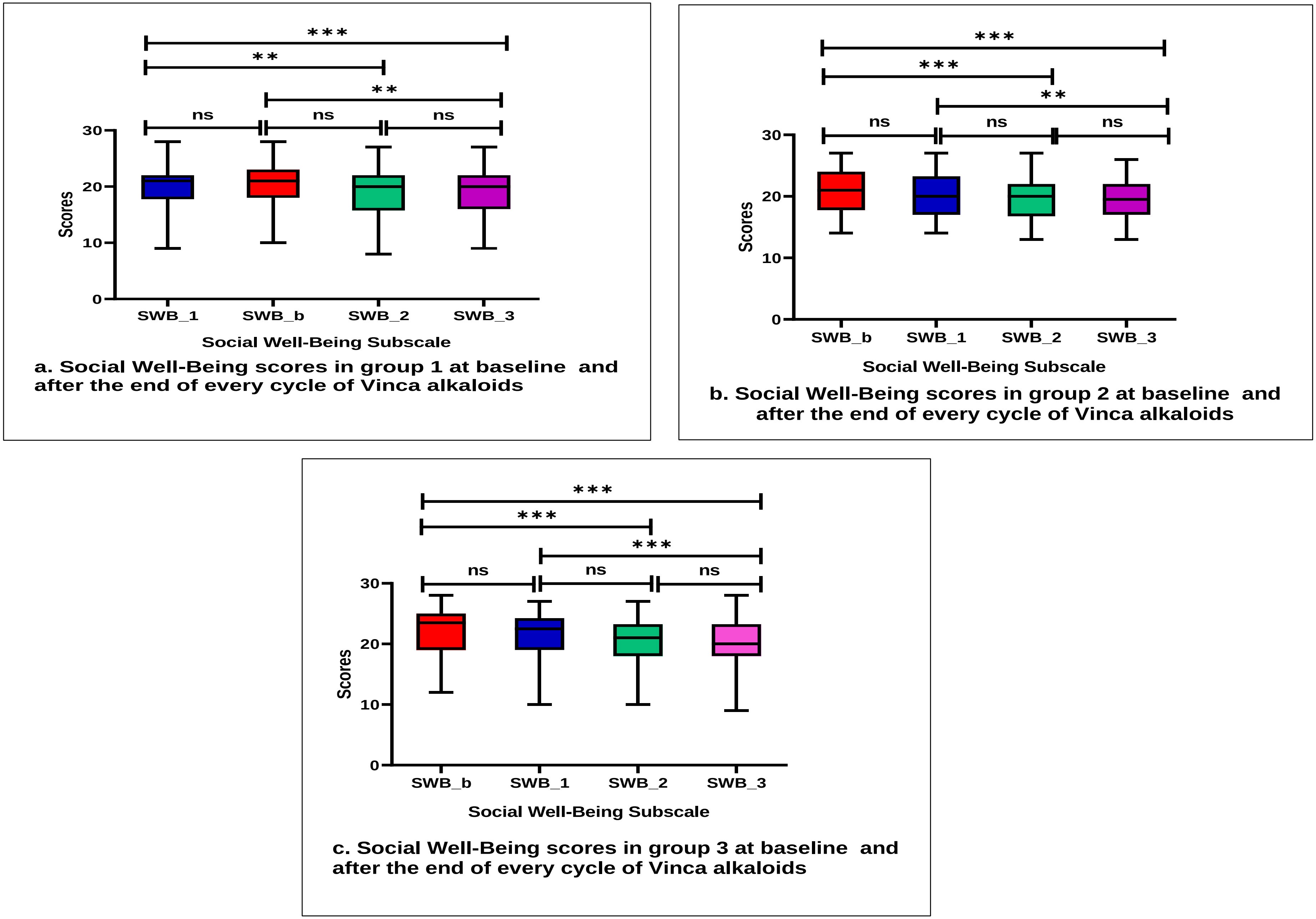

Figure S3. Social well-being subscale scores in the three groups at baseline and at the end of every cycle of the three cycles of vinca alkaloids.

Pairwise comparisons were done utilizing Dunn`s correction, data are expressed as median and IQR, ns: not statistically significant, ✱: $p<0.05$, ✱✱: $p<0.01$, ✱✱✱: $p<0.001$.

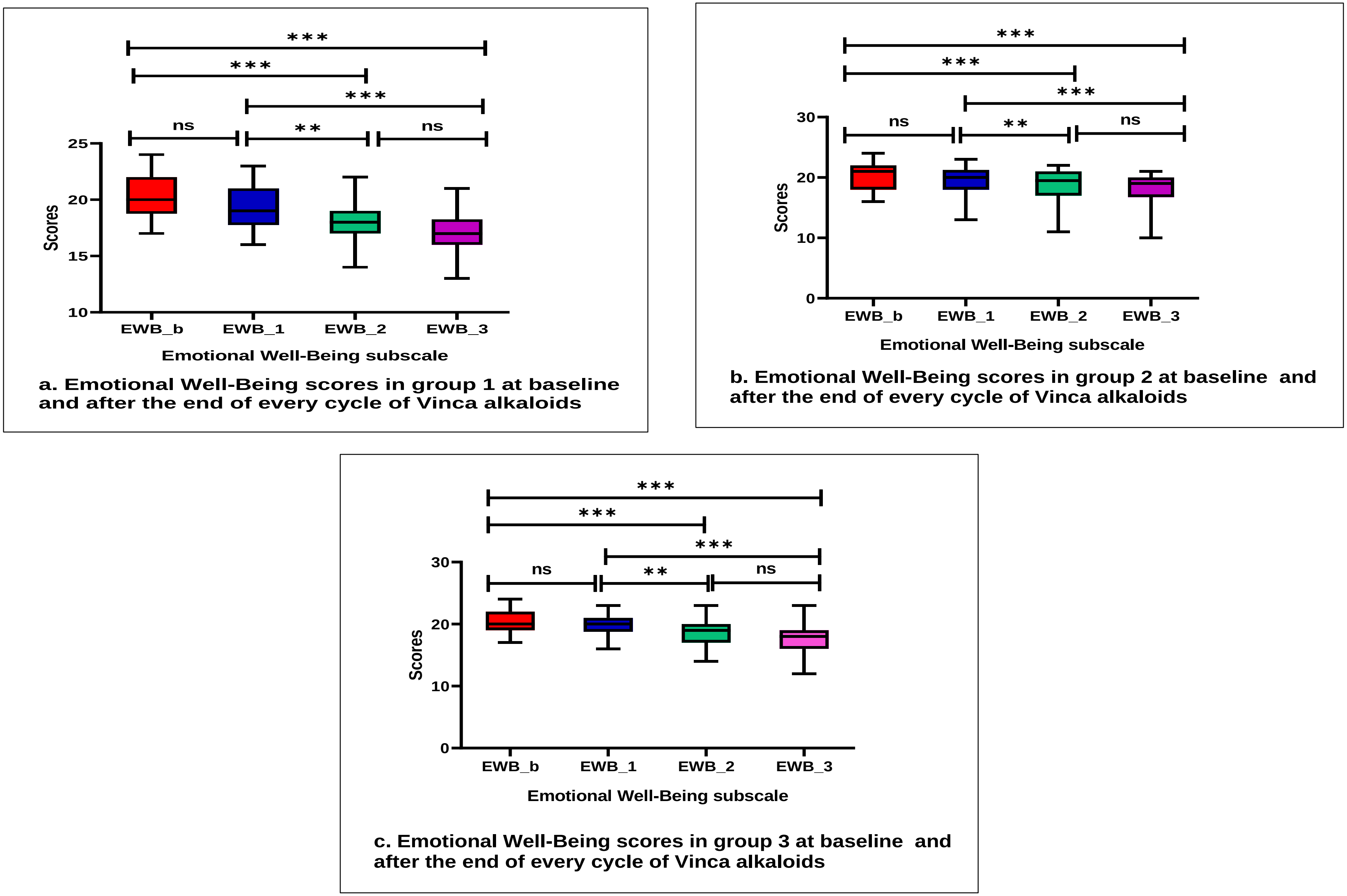

a. Emotional Well-Being scores in group 1 at baseline and after the end of every cycle of Vinca alkaloids

b. Emotional Well-Being scores in group 2 at baseline and after the end of every cycle of Vinca alkaloids

c. Emotional Well-Being scores in group 3 at baseline and after the end of every cycle of Vinca alkaloids

Figure S4. Emotional well-being subscale scores in the three groups at baseline and at the end of every cycle of the three cycles of vinca alkaloids.
Pairwise comparisons were done utilizing Dunn`s correction, data are expressed as median and IQR, ns: not statistically significant, ✱: p<0.05, ✱✱:p<0.01, ✱✱✱:p<0.001.

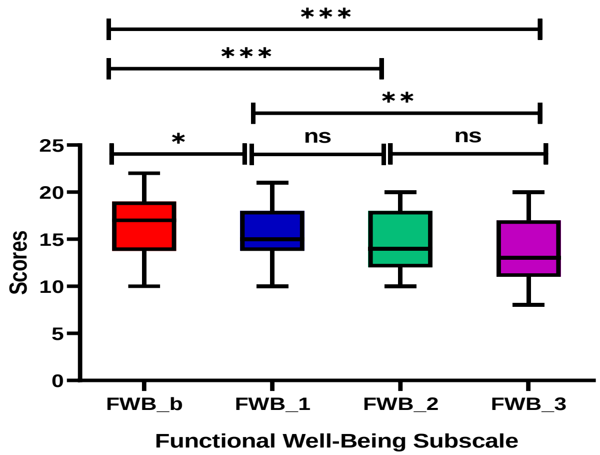

a. Functional Well-Being subscale scores in group 1 at baseline and at the end of every cycle of Vinca alkaloids

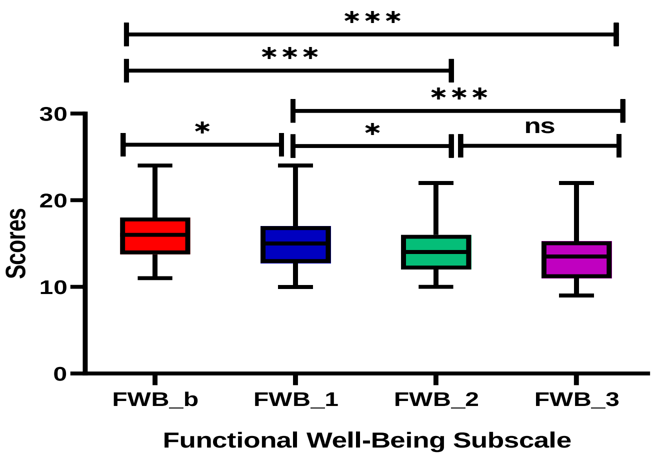

b. Functional Well-Being scores in group 2 at baseline and after the end of every cycle of Vinca alkaloids

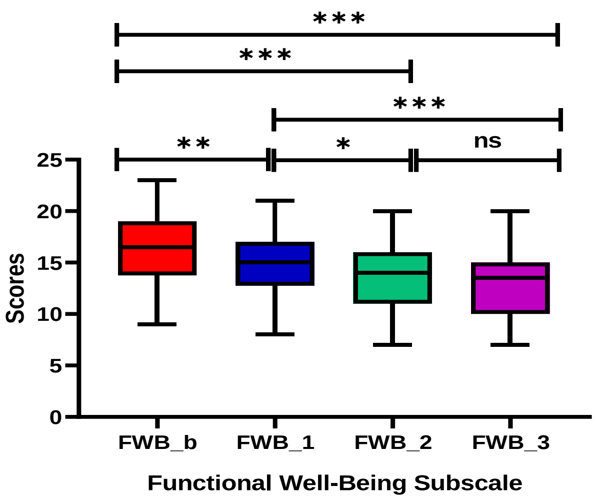

c. Functional Well-Being subscale scores in group 3 at baseline and at the end of every cycle of Vinca alkaloids

Figure S5. Functional well-being subscale scores in the three groups at baseline and at the end of every cycle of the three cycles of vinca alkaloids.

Pairwise comparisons were done utilizing Dunn`s correction, data are expressed as median and IQR, ns: not statistically significant, ✱: p<0.05, ✱✱:p<0.01, ✱✱✱:p<0.001.

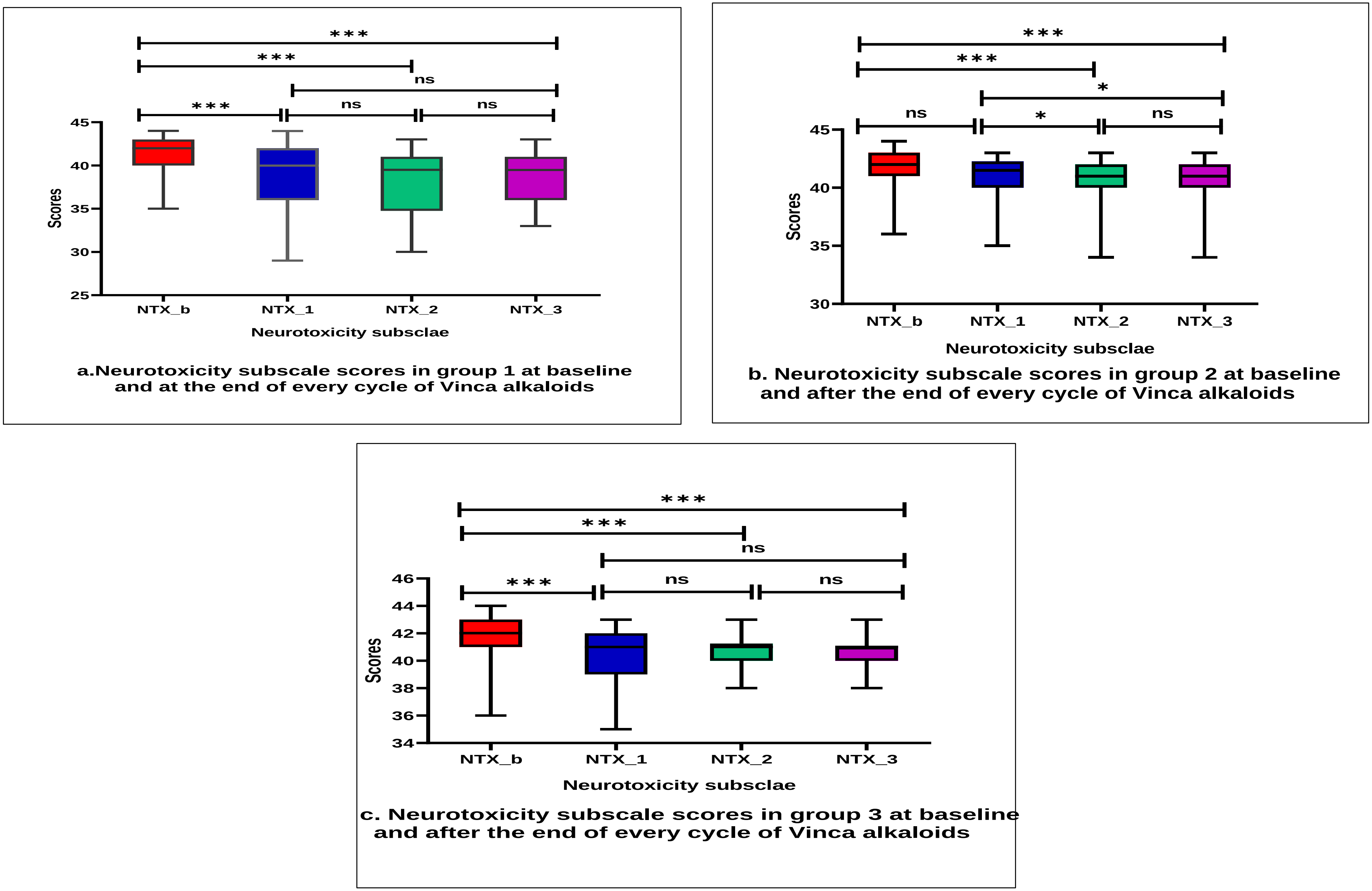

Figure S6. Neurotoxicity subscale scores in the three groups at baseline and at the end of every cycle of the three cycles of vinca alkaloids. Pairwise comparisons were done utilizing Dunn`s correction, data are expressed as median and IQR, ns: not statistically significant, ✱: $p<0.05$, ✱✱: $p<0.01$, ✱✱✱: $p<0.001$.

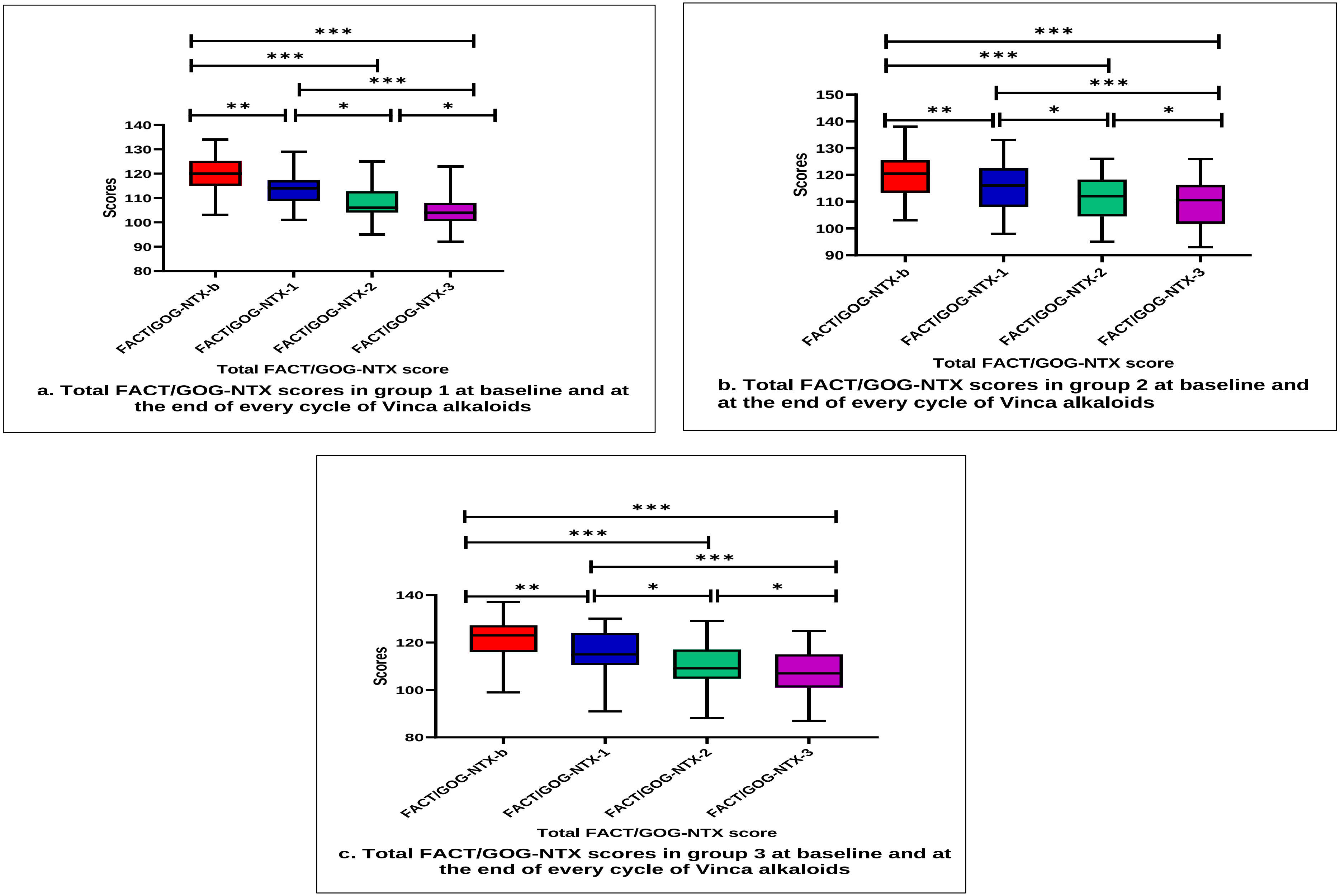

a. Total FACT/GOG-NTX scores in group 1 at baseline and at the end of every cycle of Vinca alkaloids

b. Total FACT/GOG-NTX scores in group 2 at baseline and at the end of every cycle of Vinca alkaloids

c. Total FACT/GOG-NTX scores in group 3 at baseline and at the end of every cycle of Vinca alkaloids

Figure S7. FACT/GOG NTx total scores in the three groups at baseline and at the end of every cycle of the three cycles of vinca alkaloids. Pairwise comparisons were done utilizing Dunn`s correction, data are expressed as median and IQR, ns: not statistically significant, ✱: $p<0.05$, ✱✱: $p<0.01$, ✱✱✱: $p<0.001$.

Timing of adverse drug effects:

Cox Regression analysis pairwise comparisons of paresthesia showed increase in the risk of developing paresthesia in group 1 compared to group 2 (*p*=0.011, HR 0.2, 95 % CI (0.058-0.691)), higher risk in group 1 compared to group 3 (*p* =0.007, HR 0.365, 95 % CI (0.175-0.764)) but no significant difference between group 2 and group 3 (*p*=0.657, HR 0.667, 95 % CI (0.111-3.99)).

Cox Regression analysis Pairwise comparisons of dysuria showed no difference between group 1 and group 2 (*p* =0.099, HR 0.333, 95 % CI (0.09-1.231)), between group 1 and group 3 (*p*=0.054, HR 0.471, 95 % CI (0.219-1.014)) or between group 2 and group 3 (*p*=0.657, HR 0.667, 95 % CI (0.111-3.99)). However, the risk of developing dysuria was higher in group 1 than in group 2 and group 3

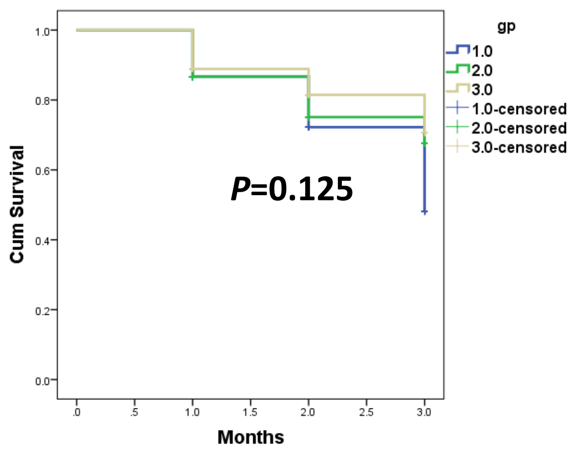

a. Cumulative survival comparison between the three groups with constipation as the event of interest.

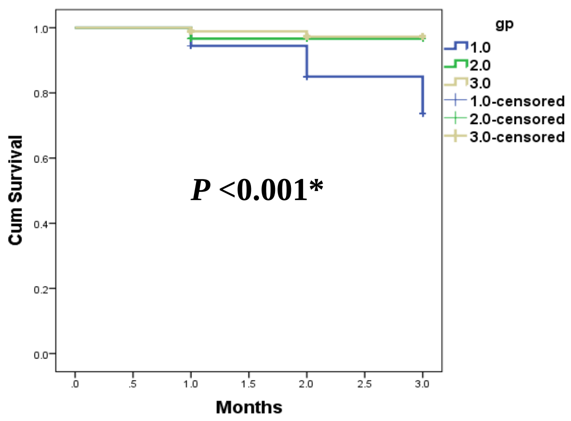

b. Cumulative survival comparison between the three groups with Paresthesia as the event of interest.

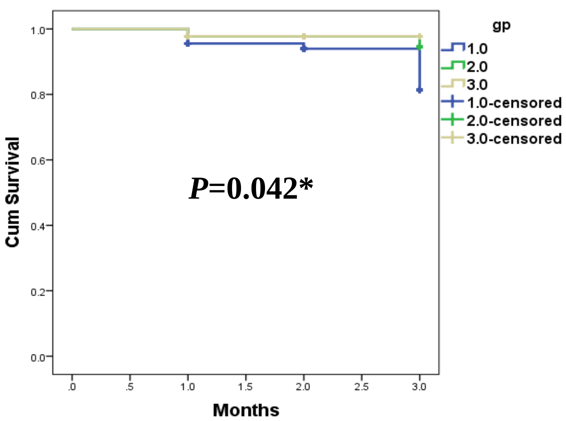

c. Cumulative survival comparison between the three groups with Dysuria as the event of interest.

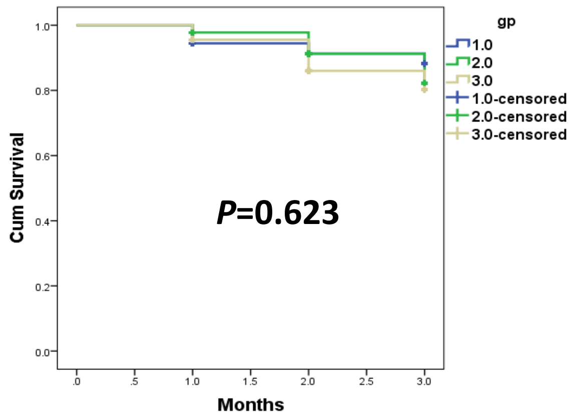

d. Cumulative survival comparison between the three groups with abdominal pain as the event of interest.

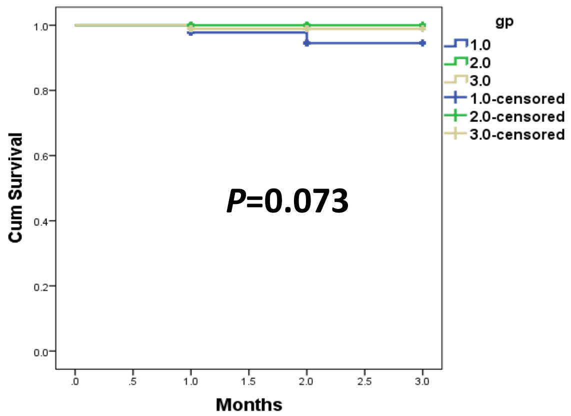

e. Cumulative survival comparison between the three groups with myalgia as the event of interest.

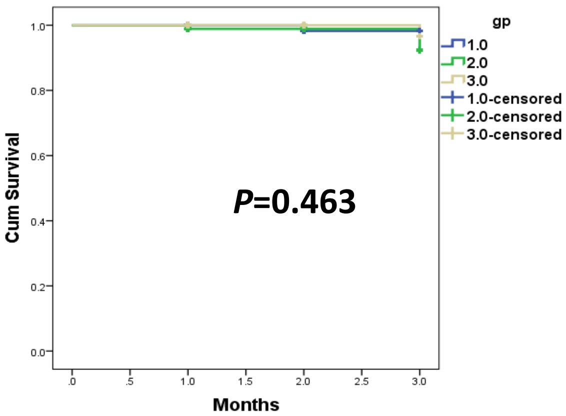

f. Cumulative survival comparison between the three groups with blurred vision as the event of interest.

Figure S8. Cumulative survival comparisons between the three groups with constipation, paresthesia, dysuria, abdominal pain, myalgia, and blurred vision as the event of interest.

Log rank *p*-values was computed using Kaplan Meier analysis, time is expressed in months *p*-values<0.05 are considered statistically significant.

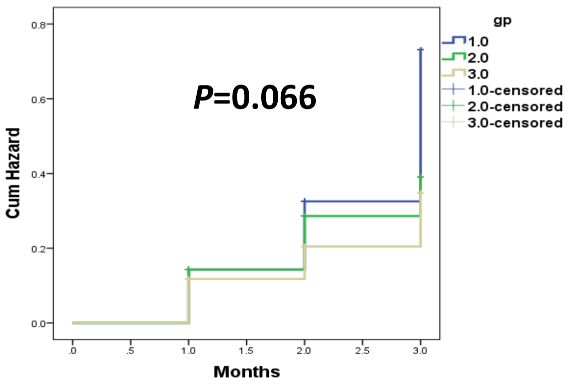

a. Cumulative hazard comparison between the three groups with constipation as the event of interest.

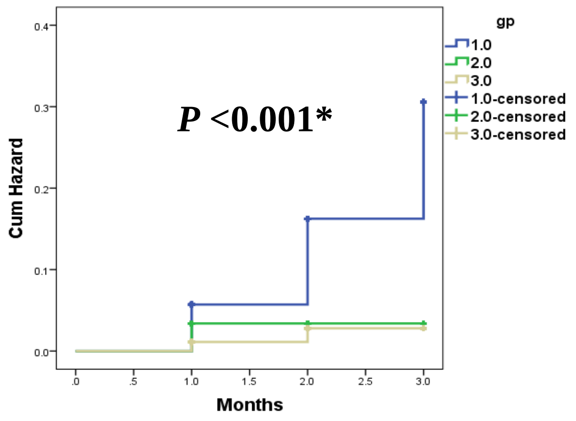

b. Cumulative hazard comparison between the three groups with Paresthesia as the event of interest.

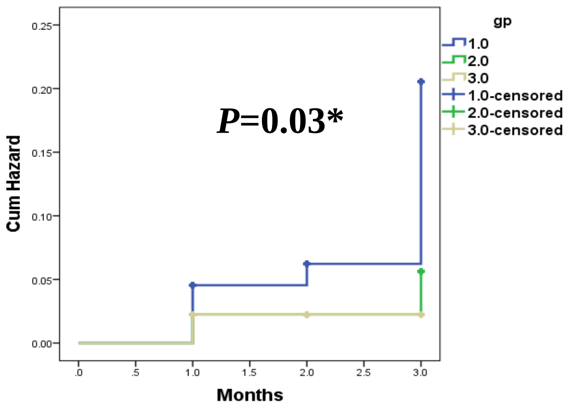

c. Cumulative hazard comparison between the three groups with Dysuria as the event of interest.

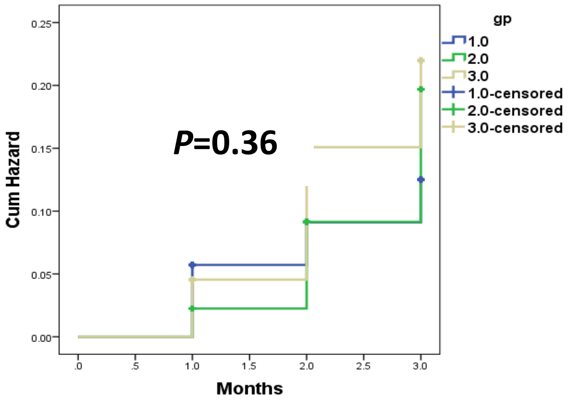

d. Cumulative hazard comparison between the three groups with Abdominal Pain as the event of interest.

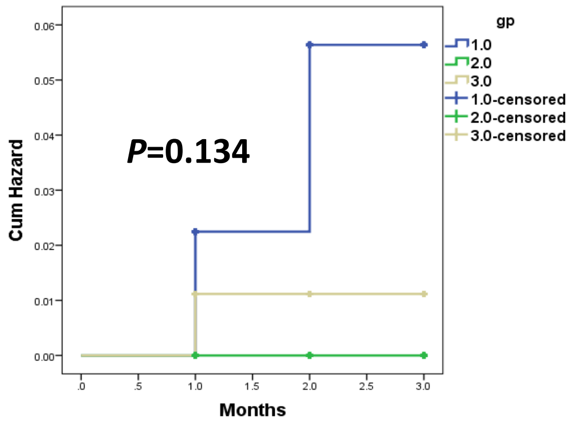

e. Cumulative hazard comparison between the three groups with myalgia as the event of interest.

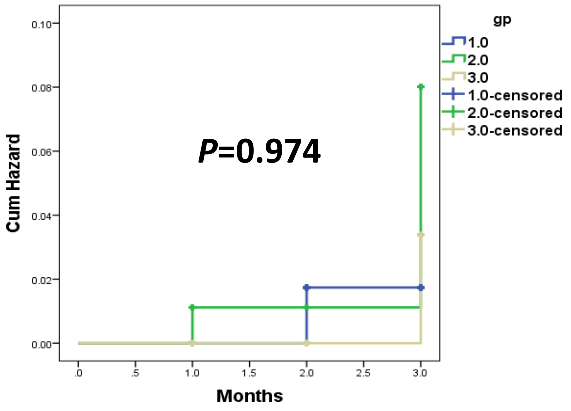

f. Cumulative hazard comparison between the three groups with blurred vision as the event of interest.

Figure S9. Cumulative hazard comparisons between the three groups with constipation, paresthesia, dysuria, abdominal pain, myalgia, and blurred vision as the event of interest.

Cox regression analysis was utilized for calculation of *p*-values, time is expressed in months *p*-values<0.05 are considered statistically significant.

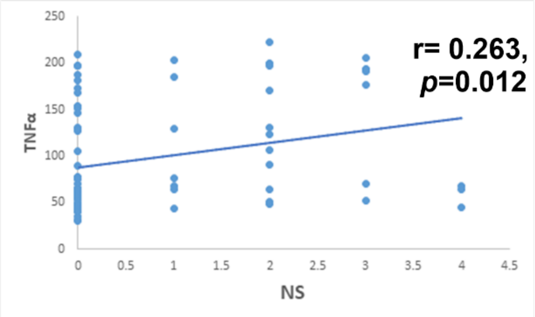

a. Correlation between NS and TNFα levels

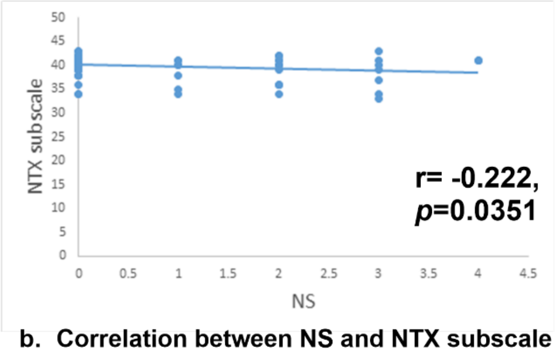

b. Correlation between NS and NTX subscale in FACT/GOG-Ntx score

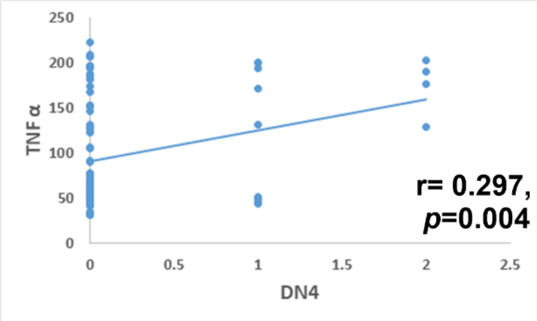

c. Correlation between DN4 and TNFα levels

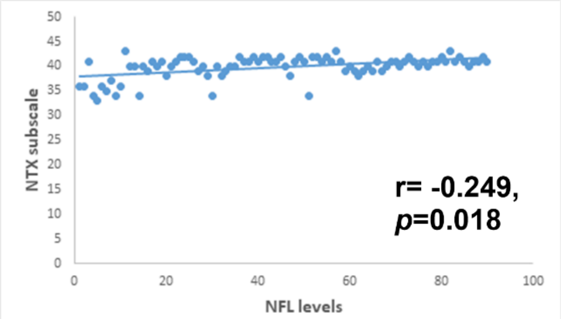

d. Correlation between NFL levels and NTX subscale in FACT/GOG-Ntx score

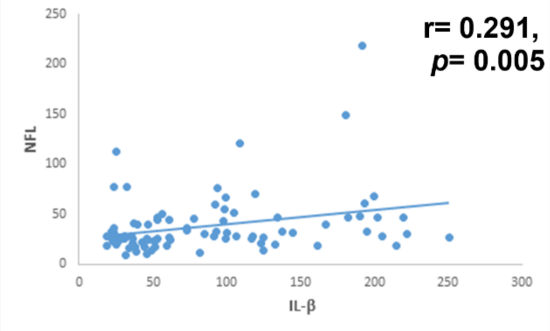

e.Correlation between NFL and IL 1-β levels after the third cycle of vinca alkaloids

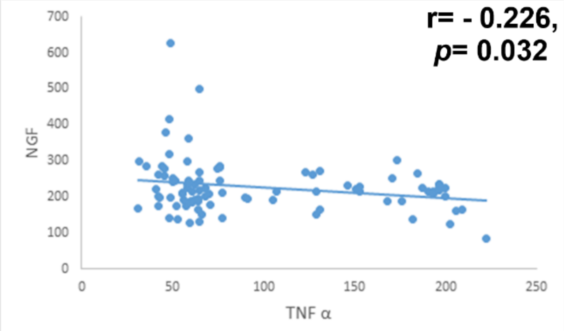

f. Correlation between NGF and TNF-α levels after the third cycle of vinca alkaloids

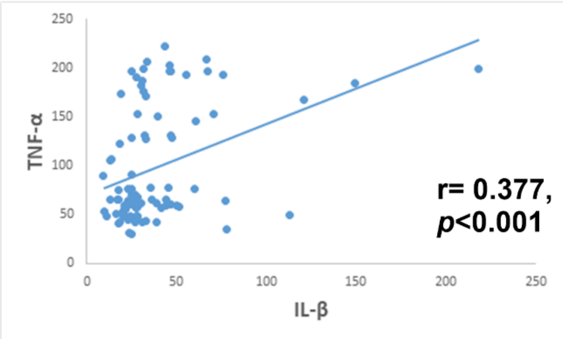

g. Correlation between TNF-α and IL 1-β levels after the third cycle of vinca alkaloids

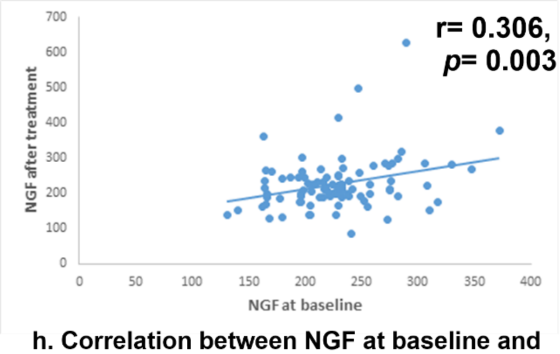

h. Correlation between NGF at baseline and NGF levels after the third cycle of vinca alkaloids

Figure S10. Considerable correlations of neuropathy scores and serum biomarkers.

References

1. Sandler, A.; Gordon, M.; De Alwis, D.P.; Pouliquen, I.; Green, L.; Marder, P.; Chaudhary, A.; Fife, K.; Battiato, L.; Sweeney, C.; et al. A Phase I trial of a potent P-glycoprotein inhibitor, zosuquidar trihydrochloride (LY335979), administered intravenously in combination with doxorubicin in patients with advanced malignancy. *Clin. Cancer Res.* **2004**, *10*, 3265-3272, doi:10.1158/1078-0432.Ccr-03-0644.
2. Tidefelt, U.; Juliusson, G.; Elmhorn-Rosenborg, A.; Peterson, C.; Paul, C. Increased intracellular concentrations of doxorubicin in resistant lymphoma cells in vivo by concomitant therapy with verapamil and cyclosporin A. *Eur. J. Haematol.* **1994**, *52*, 276-282, doi:10.1111/j.1600-0609.1994.tb00096.x.
3. Ali, N.; AlAsmari, A.F.; Imam, F.; Ahmed, M.Z.; Alqahtani, F.; Alharbi, M.; AlSwayyed, M.; AlAsmari, F.; Alasmari, M.; Alshammari, A.; et al. Protective effect of diosmin against doxorubicin-induced nephrotoxicity. *Saudi J. Biol. Sci.* **2021**, *28*, 4375-4383, doi:https://doi.org/10.1016/j.sjbs.2021.04.030.
4. Santos, S.A.d. Protective activity of diosmin on doxorubicin-induced cardiotoxicity in mice with sarcoma. Federal University of Sergipe, 2022.
5. AlAsmari, A.F.; Alharbi, M.; Alqahtani, F.; Alasmari, F.; AlSwayyed, M.; Alzarea, S.I.; Al-Alallah, I.A.; Alghamdi, A.; Hakami, H.M.; Alyousef, M.K.; et al. Diosmin Alleviates Doxorubicin-Induced Liver Injury via Modulation of Oxidative Stress-Mediated Hepatic Inflammation and Apoptosis via NfkB and MAPK Pathway: A Preclinical Study. *Antioxidants* **2021**, *10*, 1998.
6. Madani, B.; Burzangi, A.; Alkreathy, H.; Karim, S.; Shaik, R.A.; Khan, L. Thymoquinone Prevents Doxorubicin-induced Hepatic-injury by Mitigating the Impairment of Mitochondrial Respiration and Electron Transport. *International Journal of Pharmaceutical Research & Allied Sciences* **2022**, *11*.
7. Musyayyadah, H.; Wulandari, F.; Nangimi, A.F.; Anggraeni, A.D.; Meiyanto, E. The growth suppression activity of diosmin and PGV-1 co-treatment on 4T1 breast cancer targets mitotic regulatory proteins. *Asian Pacific Journal of Cancer Prevention: APJCP* **2021**, *22*, 2929.
